# A Dynamic Gene Regulatory Network Model That Recovers the Cyclic Behavior of *Arabidopsis thaliana* Cell Cycle

**DOI:** 10.1371/journal.pcbi.1004486

**Published:** 2015-09-04

**Authors:** Elizabeth Ortiz-Gutiérrez, Karla García-Cruz, Eugenio Azpeitia, Aaron Castillo, María de la Paz Sánchez, Elena R. Álvarez-Buylla

**Affiliations:** 1 Instituto de Ecología, Universidad Nacional Autónoma de México, 3er Circuito Exterior, Junto a Jardín Botánico Exterior, México, D.F. CP 04510, México; 2 Centro de Ciencias de la Complejidad-C3, Universidad Nacional Autónoma de México, Ciudad Universitaria, Apartado Postal 70–275, México, D.F. 04510, México; UNITED STATES

## Abstract

Cell cycle control is fundamental in eukaryotic development. Several modeling efforts have been used to integrate the complex network of interacting molecular components involved in cell cycle dynamics. In this paper, we aimed at recovering the regulatory logic upstream of previously known components of cell cycle control, with the aim of understanding the mechanisms underlying the emergence of the cyclic behavior of such components. We focus on *Arabidopsis thaliana*, but given that many components of cell cycle regulation are conserved among eukaryotes, when experimental data for this system was not available, we considered experimental results from yeast and animal systems. We are proposing a Boolean gene regulatory network (GRN) that converges into only one robust limit cycle attractor that closely resembles the cyclic behavior of the key cell-cycle molecular components and other regulators considered here. We validate the model by comparing our *in silico* configurations with data from loss- and gain-of-function mutants, where the endocyclic behavior also was recovered. Additionally, we approximate a continuous model and recovered the temporal periodic expression profiles of the cell-cycle molecular components involved, thus suggesting that the single limit cycle attractor recovered with the Boolean model is not an artifact of its discrete and synchronous nature, but rather an emergent consequence of the inherent characteristics of the regulatory logic proposed here. This dynamical model, hence provides a novel theoretical framework to address cell cycle regulation in plants, and it can also be used to propose novel predictions regarding cell cycle regulation in other eukaryotes.

## Author Summary

In multicellular organisms, cells undergo a cyclic behavior of DNA duplication and delivery of a copy to daughter cells during cell division. In each of the main cell-cycle (CC) stages different sets of proteins are active and genes are expressed. Understanding how such cycling cellular behavior emerges and is robustly maintained in the face of changing developmental and environmental conditions, remains a fundamental challenge of biology. The molecular components that cycle through DNA duplication and citokinesis are interconnected in a complex regulatory network. Several models of such network have been proposed, although the regulatory network that robustly recovers a limit-cycle steady state that resembles the behavior of CC molecular components has been recovered only in a few cases, and no comprehensive model exists for plants. In this paper we used the plant *Arabidopsis thaliana*, as a study system to propose a core regulatory network to recover a cyclic attractor that mimics the oscillatory behavior of the key CC components. Our analyses show that the proposed GRN model is robust to transient alterations, and is validated with the loss- and gain-of-function mutants of the CC components. The interactions proposed for *Arabidopsis thaliana* CC can inspire predictions for further uncovering regulatory motifs in the CC of other organisms including human.

## Introduction

The eukaryotic cell cycle (CC) in multicellular organisms is regulated spatio-temporally to yield normal morphogenetic patterns. In plants, organogenesis occurs over the entire lifespan, thus CC arrest, reactivation, and cell differentiation, as well as endoreduplication should be dynamically controlled at different points in time and space [1]. Endoreduplication is a variation of the CC, in which cells increase their ploidy but do not divide. Normal morphogenesis thus depends on a tight molecular coordination among cell proliferation, cell differentiation, cell death and quiescence. These biological processes share common regulators which are influenced by environmental and developmental stimuli [1–3]. It would not be parsimonious to depend on different regulatory circuits to control such interlinked cellular processes, CC behaviors and responses. Thus we postulate that a common network is deployed in all of them. Such overall conserved CC network may then connect to different regulatory networks underlying cell differentiation in contrasting tissue types or to signal transduction pathways elicited under different conditions, and thus yield the emergence of contrasting cellular behaviors in terms of cycling rate, entrance to endocycle, differentiation, etc.

Furthermore, the overall CC behaviors are widely conserved and robust among plants and animals. Hence, we aim at further investigating the collective behavior of the key upstream regulators and studied CC components to understand the mechanisms involved in the robustness of CC regulation under changing developmental stages and environmental conditions faced by plants along their life-cycles. Previous studies, that have shown the oscillatory behavior of several transcription factors, that had not been associated as direct regulators of the CC, support our proposed hypothesis [4]. We thus propose to uncovering the set of necessary and sufficient regulatory interactions underlying the core regulatory network of plant CC, including some key upstream transcriptional regulators.

Computational tools are essential to understanding the collective and dynamical behavior of these components within the regulatory networks involved. As a means of uncovering the main topological and architectural traits of such networks, we propose to use Boolean formalisms that are simple and have proven to be useful and powerful to follow changes in the activity of regulators of complex networks in different organisms and biological processes [5, 6].

Although the key CC components have been described in different organisms, the complexity and dynamic nature of the molecular interactions that are involved in CC regulation and the emergence of the cyclic behavior of the CC molecular components are not well understood yet. The use of systemic, dynamic and mathematical or computational approaches has been useful towards this already. Previous models have focused mainly on yeast and animal systems and have been useful to analyze many traits of CC behavior such as robustness, hysteresis, irreversibility and bistability [7–11]. The latter two properties have been validated with experimental data [12–14].

We herein summarize the main traits and components of the eukaryotic CC. The molecular CC regulators have been described and they are well conserved across distantly related organisms [15, 16]. CC progression is regulated by Cyclin-Dependent Kinases (CDKs) [17] that associate with different cyclins to confer substrate specificity [18]. CDK-cyclin complexes trigger the transition from G1 (Gap 1) to synthesis phase (S phase) in where the genome is duplicated, and from G2 (Gap 2) to mitotic phase (M phase) for the delivery of the newly duplicated DNA to the two daughter cells [19] (see for a review [17, 20]). The CDK-cyclin activity also regulates the cell transit between G and S phases during the endoreduplication process [21, 22].

Two CDKs (CDKA and CDKB) are involved in CC regulation. CDKA;1-CYCDs and CDKA;1-CYCA3 complexes regulate G1/S and S phase progression [23–25]; while CDKB-CYCA2 and CDKB-CYCBs regulate G2/M phase and M progression [26–28]. Thus CDK-cyclin activity is finely-tuned by phosphorylation, interactions with CDK inhibitors such as Kip-related proteins (KRPs), and degradation of cyclins and KRPs by Skp1/Cullin/F-box (SCF), as well as by the anaphase-promoting complex/Cyclosome (APC/C) [29–31]. Besides these components, plant CC machinery has a greater number of CC regulators than other eukaryotes and some of those components such as the CDKB are plant-specific.

Several key transcriptional regulators participate in the G1/S and G2/M transitions [32]. The E2F/RBR pathway regulates G1/S transition by transcriptional modulation of many genes required for CC progression and DNA replication [33, 34]. While E2Fa and E2Fb with their dimerization partner (DP) activate transcription of a subset of S phase genes, E2Fc-DP represses transcription [35]. The function of E2Fa and E2Fb is inhibited by their interaction with RBR [36]; in G1/S transition CDKA;1-CYCD-mediated RBR hyperphosphorylation, releases E2Fa/b-DP heterodimers allowing transcriptional activation of E2Fa and E2Fb targets. Simultaneously the E2Fc-DP transcriptional inhibitor is degraded [37].

Little is known about the regulation of G2/M transition in plants, however a class of conserved transcription factors belonging to the MYB family has been described, that seem to have key roles in CC regulation. MYB transcription factors have a prominent role during G2/M transition, by regulating, for example, CYCB1;1 which is determinant in triggering mitosis [38–43]. For the mitosis exit, APC/C mediates degradation of the mitotic cyclins as CYCB1;1 and CYCA2;3, inactivating CDK-cyclin complexes. CCS52A2, an activator subunit of APC/C, is transcriptionally inhibited by E2Fe [44].

Some previous models have recovered the limit cycle attractor as well for CC components [45–48]. A pioneer model of the CC focused on mitotic CDK-cyclin heterodimer and a cyclin protease oscillatory behavior [49]. On the other hand, Novak and Tyson incorporated additional nodes and interactions to model the G1/S and G2/M transitions of the *S. pombe* CC [50, 51]. They also analyzed evolutionary roles of CC regulators [52], mutant phenotypes [53], stable steady states [7] and the role of cues such as cell size or pheromones in CC progression [54, 55]. Additionally, comprehensive CC continuous models [45] and generic modules for eukaryotic CC regulation [56, 57] have been proposed.

In addition to continuous formalisms, CC models have used discrete approaches as Boolean models for yeast and mammalian systems [46–48, 58–61], and more recently, hybrid models for mammalian cells have been published [62]. Subsequently, time-delayed variables [63] and variables defining CC events [47, 48] were incorporated. Time robustness was improved with specifications of the temporal order with which each component is activated [60]. Recent published reports on CC dynamics use steady state probability distributions and potential landscapes, and highlight the enormous potential of CC models to characterize normal and altered regulation of mammalian CC [64, 65].

Yeast CC Boolean models with summatory thresholds [58, 59], incorporated self-degradation for proteins, but did not incorporate several negative regulators explicitly. In a later work [61], nodes were kept active when the summatory effect of their regulators was greater than the activation threshold, which implies self-degradation of the protein, when such summatory is equal to or below the threshold. Fauré and Thieffry have transformed CC Boolean models, that use threshold functions, to models with a combinatorial scheme, and they have also presented a broader discussion about these two approaches to logical frameworks [66].

Two Boolean models of budding yeast CC and another one of mammalian CC recover cyclic attractors [46–48]. The mammalian CC model [46] also recovers a fixed-point attractor corresponding to G0. In another study, Fauré and collaborators integrated three modules to yield a comprehensive model for the budding yeast CC GRN [47]. The components included variables to represent cellular growth, citokinesis, bud formation, DNA replication and the formation of the spindle. The yeast CC model by Irons also included variables of CC events (e.g. bud formation or DNA replication) as well as time delays [48]. In contrast to other eukaryotes, in *Arabidopsis thaliana* (*A. thaliana* herein) very few attempts have been made to integrate available experimental data on CC regulators using mechanistic models. Only a study that considers the G1/S transition has been proposed and contributed to show some additional conserved features of this CC control point among eukaryotes [67].

We integrated available experimental data on 29 *A. thaliana* regulatory interactions involved in CC progression into a Boolean discrete model, that recovers key properties of the observed plant CC. The regulatory network, that we put forward, also incorporates three uncovered interactions, based on animal systems (E2Fb → SCF, CDKB1;1-CYCA2;3 ⊣ E2Fa, APC/C ⊣ SCF), as well as 16 interactions based on bioinformatic analyses. Therefore, the latter proposed interactions constitute new predictions that should be tested experimentally. The use of yeast or animal data is supported by the fact that main CC components or regulatory motifs are conserved among eukaryotes [16]. In our model, we include solely molecular components and avoid artificial self-degradation loops, which have been used for recovering the limit cycle attractor. We validated the model simulating loss- and gain-of-function lines, and hence demonstrate that the Boolean network robustly implements true dynamical features of the biological CC regulatory network under wild type and genetic alterations. Possible artifacts due to the discrete dynamical nature of the model used, and of its synchronous updating scheme, were discarded by comparing the Boolean model results to those of a continuous approximation model. The continuous model indeed recovers the robust limit cycle that mimics the dynamical behavior of CC components under a wide range of parameters tested. Finally, we provide novel predictions that can be tested against biological experimental measurements in future studies. The model put forward constitutes a first mechanistic and integrative explanation to *A. thaliana* CC.

## Materials and Methods

### Boolean model

We proposed a Boolean approach to integrate and study the qualitative complex logic of regulation of the molecular components underlying the CC dynamics. We formalized available experimental data on logical functions and tables of truth that rule how the state of a particular component is altered as a function of the states of all the components that regulate it. In a Boolean model each node state can be 0, when the expression of a gene or other type of molecular component or complex of such components is unexpressed or “OFF”, or 1 when it is expressed, or “ON”. Nodes states are updated according to the function: *X*_*i*_(*t*+1) = *F*_*i*_(*X*_*i*_1__(*t*), *X*_*i*_2__(*t*), …, *X*_*i*_*k*__(*t*)), where *X*_*i*_(*t*+1) is the state of *X*_*i*_ gene at time *t*+1 and *X*_*i*_1__(*t*), *X*_*i*_2__(*t*), …, *X*_*i*_*k*__(*t*) is the set of its regulators at time *t*. The set of logical rules for all the network components defines the dynamics of the system. By applying the logical rules to all nodes for several iterations, the dynamics of the whole network can be followed until it reaches a steady state; a configuration or set of configurations that does not change any more or are visited in a cyclical manner, respectively. Such state is called an “attractor”. Single-point attractors only have one GRN configuration, or cyclic attractors with period *n*, which have *n* configurations that are visited indefinitely in the same order. In this paper we propose a GRN model that converges to a single limit cycle attractor that recovers the CC molecular components’ states of presence (network configuration) in a cyclic pattern that mimics the pattern observed for the molecular components included in the model along the different CC phase.

### Model assumptions

*A. thaliana* CC Boolean model has the following assumptions:

1. Nodes represent mRNA, proteins or protein complexes involved in CC phase transitions. Node state “ON” is for the presence of regulator, and “OFF” is for absence; in the latter case, it may also indicate instances in which a component may be present but non-functional due to a post-translational modification.
2. The state of the RBR (RETINOBLASTOMA-RELATED) node corresponds to a 1 or “ON” when this protein is in its hypo-phosphorylated form and therefore is ready to inhibit E2F transcription factors.
3. When a particular CDK is not specified, a cyclin can form a complex with CDKA;1, a kinase that is always present because it is expressed in proliferative tissues [68] during the complete CC.
4. E2Fa, E2Fb and E2Fc need dimerization partner proteins (DPa or DPb) for its DNA-binding. Given that DP expression does not change drastically in CC [69], we assumed that the state of these heterodimers is given only by the presence of E2F factors.
5. The Boolean logical functions integrate and formalize experimental data available mainly for the *A. thaliana* root apical meristem, however some data from leaves were considered, and we assumed that these are also valid for CC regulation in the root meristem. Also, data from other systems and data obtained by sequence promoter analysis were considered as indicated in each case [27, 39, 40, 67, 70–85] (summarized in Table 1).
6. The dynamics of complex formation (such as CDK-cyclin and KRP1, or RBR and E2F factors) are specified directly in the Boolean function of their target genes. For instance, the logic rule for E2Fb is *E2Fa* & *!RBR*, indicating that E2Fb state is “ON” when it is transcriptionally activated by E2Fa free of RBR. All E2Fa targets also included in their logical rules RBR, as is shown in S1 Text. Then, the presence of KRP1 or RBR in a logical rule does not imply that they are regulators acting directly on the corresponding target.
7. The updating scheme for the node states was synchronous.

**Table 1:** Hypothetical Interactions for the *A. thaliana* CC Network.

Regulator		Target	Data supporting the proposition of the interaction	Refs.
**E2Fb**	→	SCF	F-box protein Skp2 is part of the SCF complex and is transcriptionally regulated by E2F1 in humans. In *A. thaliana*, it has only been reported that E2F factors regulate FBL17, another F-box protein.	[67, 70]
**E2Fb**	→	MYB77	Direct regulation between E2F and MYB factors has been reported in budding yeast and mammals, but in plants it could include at least one intermediary; *A. thaliana* could have a similar regulation because its CC also presents transcriptional waves in G1/S and G2/M transitions as yeasts and mammals. After analyzing the two main families of transcription factors involved in CC regulation: TCP and MYB, we propose MYB77 as a mediator between E2F and MYB regulation. Using available microarray analyses, we found that MYB77 shows CC-dependent expression with a peak in M phase. In addition to having binding sites for E2F, with the identification of the binding site recognized by MYB77, we can hypothesize that MYB77 regulates MYB3R1/4 and other CC genes.	[39, 71–74]
**MYB77**	→	E2Fe, KRP1, MYB3R1/4, CYCB1;1, CYCA2;3, CDKB1;1, CCS52A2	The sequence CNGTTR identified as a consensus site recognized by MYB77 was used to find its possible targets among CC core genes. Several of them are expressed just before G2 to M phase transition.	[75–77]
**MYB3R1/4**	→	SCF, RBR, CDKB1;1, CYCA2;3, APC/C, E2Fc, MYB3R1/4, KRP1	The consensus site of MYB3R4 was found in SKP2A, RBR, CDKB1;1, CYCA2;3, CCS52A2, KRP1, E2Fc, MYB3R1/4 and CYCB1;1 by *in silico* analysis described in the Materials and Methods section. In tobacco, NtmybA1 and NtmybA2 genes have the MSA sequence and they can regulate themselves. MYB3R1/4 might promote the expression of KRP1, since KRP1 has a peak of expression in G2/M and has eight putative MSA elements. CYCB1;1 regulation by MYB3R1/4 also has experimental support.	[40, 78]
**CDKB1;1-CYCA2;3**	⊣	E2Fa	It has been hypothesized that a cause of low levels of E2Fa could be due to its high turnover rate as result of CDKB1;1 phosphorylation. This E2F factor has putative CDK-phosphorylation sites in its N-terminal end, and a high CDK activity inversely correlates with its DNA binding ability *in vitro*. This hypothesis is supported by observations in mammalian cells.	[27, 79–81]
**APC/C**	⊣	SCF	It was proposed that APC/C and SCF functions are mutually exclusive during CC progression, which led to the identification of the relationship amongst them. In proliferating mammal cells, levels of Skp2, a SCF subunit, oscillate under the pattern of APC/C substrates. Furthermore, the APC/C subunit Cdh1 participates in the degradation of Skp2 and the reduction of Cdh1 expression stabilizes Skp2. *A. thaliana* SCF and APC/C seem have the same roles during CC as their animal counterparts.	[82–85]

A summary of the data led us to propose interactions that have not been previously described for *A. thaliana* CC. ⊣ stands for negative regulation and → for positive regulation.

### Periodic expression and promoter sequence analysis

Most regulatory interactions and logical rules were obtained from the *A. thaliana* data [20, 21, 25–27, 29, 30, 35, 37, 38, 40, 43, 44, 78–80, 85–103] (detailed in Table 2). *A. thaliana* CC-dependent expression data for validation was obtained from: [72–74]. The consensus site used for MYB77 was CNGTTR, according to: [75, 76], while that for MYB3R4 was AACGG according to: [43]. The motifs were searched in the regulatory sequences of all network nodes using Pathmatch tool (http://arabidopsis.org/cgi-bin/patmatch/nph-patmatch.pl) of TAIR. Regulatory sequences in TAIR10 Loci Upstream Sequences-1000bp and TAIR10 5’ UTRs datasets were used.

**Table 2:** Experimental Interactions for the *A. thaliana* CC Network and their Evidence.

Regulator		Target	Description of the interaction	Refs.
**CDKA;1-CYCD3;1**	⊣	RBR	Studies suggest that complexes formed by CDKA;1 and D-type cyclins phosphorylate RBR.	[20, 86–89]
**CDKA;1-CYCD3;1**	⊣	RBR–E2Fb	E2Fb–RBR complex diminishes in CYCD3;1 overexpressor line.	[90]
**CDKA;1-CYCD3;1**	⊣	E2Fc	CDKA;1 bound to D-type cyclin affects formation of E2Fc-DPb complex and its binding to DNA. The recognition of E2Fc by the SCF complex depends on phosphorylation mediated by CDKA;1.	[35, 37, 91]
**SCF**	⊣	CYCD3;1	SCF is involved in the ubiquitination required for CYCD3;1 degradation.	[92]
**SCF**	⊣	KRP1	SCF ubiquitinates KRP1 to be degraded.	[85, 93]
**SCF**	⊣	E2Fc	E2Fc shows the accumulation in *skp2a* mutant (subunit of SCF); the overexpression of SKP2A reduces levels of E2Fc.	[35, 91]
**RBR**	⊣	E2Fa/b	RBR is a negative regulator of E2Fa/b transcriptional activity.	[90]
**E2Fa**	→	E2Fb	E2Fb transcription is induced in E2Fa overexpressor line.	[94]
**E2Fa**	→	E2Fc	E2Fc has binding sites for E2F and it is induced in E2Fa-DPa overexpressors.	[80, 94]
**E2Fa**	→	RBR	Transcriptional control of RBR is under E2Fa transcriptional activity.	[95]
**E2Fa**	→	APC/C	CCS52A2, a component of APC/C, is induced when RBR-free E2Fa is overexpressed.	[90]
**E2Fb**	→	CYCB1;1	CYCB1;1 expression is induced when RBR-free E2Fb increases; targets of E2Fb are genes needed for G2/M transition.	[79, 80, 90]
**E2Fb**	→	CDKB1;1	Inducible expression of E2Fb promotes CDKB1;1 expression.	[79]
**E2Fb**	→	E2Fe	E2Fb induces transcription of E2Fe.	[96]
**E2Fc**	⊣	CDKB1;1	The effect of E2Fb can be countered by E2Fc; with E2Fc destabilization increments CDKB1;1.	[96, 97]
**E2Fc**	⊣	CYCB1;1	CYCB1;1 expression increases when E2Fc expression is silenced; E2Fc overexpression reduces CYCB1;1 level.	[37]
**E2Fc**	⊣	E2Fa	E2Fa messengers increase when E2Fc expression is silenced.	[37]
**E2Fc**	⊣	E2Fe	E2Fc counteracts the positive effect that E2Fb has in the expression of E2Fe.	[96]
**E2Fe**	⊣	APC/C	Expression of CCS52A, a subunit of APC/C, is downregulated by E2Fe.	[44]
**MYB3R1/4**	→	CYCB1;1	MYB3R1/4 recognizes the sequence AACGG required for CYCB1;1 expression; other regulators seem to drive its G2/M-specific expression.	[38, 43]
**CDKB1;1**	–	CYCA2;3	CYCA2;3 interacts with CDKB1;1 to form a functional complex.	[25, 27]
**CDKB1;1-CYCA2;3**	⊣	KRP1	In complex with CYCA2;3, CDKB1;1 could promote KRP1 proteolysis as promotes KRP2 proteolysis; both KRPs could have similar roles in mitosis entry, since both interact with CDKA;1 and are expressed in G2/M.	[21, 27, 78]
**CDKB1;1, CDKA;1**	–	CYCB1;1	B-type cyclins interact with B-type and A-type CDKs.	[25, 26]
**CDKA;1-CYCB1;1**	→	MYB3R1/4	The overexpression of MYB3R4 enhances the 2-fold activity of its target promoters in comparison to WT, and the co-expression of MYB3R4 and CYCB1;1 enhances them 4-fold; CycB1 and other mitotic cyclins enhances the activity of NtmybA2 factors in tobacco.	[40, 98, 99]
**KRP1**	⊣	CYCD3;1	KRP1 is able to interact with CDKA;1 and CYCD3;1.	[29, 93, 100, 101]
**KRP1**	⊣	CYCB1;1	KRP1 binding to CDKA;1 inhibits the activity of CDKA–CYCB1;1.	[30, 100]
**APC/C**	⊣	CYCB1;1	The APC/C complex ubiquitinates CYCB1;1 to be degraded.	[102]
**APC/C**	⊣	CYCA2;3	CYCA2;3 is stabilized with loss-of-function mutations in APC/C subunits or with mutations in its D-box.	[27, 103]

Summary of experimental evidence supporting interactions of *A. thaliana* CC GRN. ⊣ represents negative regulation, → is for positive and - represents the formation of functional complex.

### Software for robustness analysis and mutant simulation

We used *BoolNet* [104] (a library of *R* language [105]) and *Atalia*(Á. Chaos; http://web.ecologia.unam.mx/achaos/Atalia/atalia.htm) to simulate the CC GRN dynamics and perform robustness, and mutant analyses. Systematic alterations in Boolean functions for robustness analyses were done with Atalia, while stochastic perturbations in random networks to compare attractor’s robustness were done with BoolNet. For random perturbations made in transitions between network configurations or in Boolean functions, the “bitflip” method was applied. To validate the GRN model proposed here, we used BoolNet and simulated loss- and gain-of-function mutations for each node, by skipping the node’s logical rule and setting the respective gene to “0” and “1”, respectively.

### Continuous model

For the continuous model, we followed [106, 107]. In the continuous version of the model the rate of change for each *x*_*i*_ node is represented by a differential equation that comprises production as well as decay rates:

$$\begin{array}{c} \frac{dx_{i}}{dt}=\frac{-e^{0.5h}+e^{-h*(\omega_{i})}}{\left(1-e^{0.5h}\right)*\left(1+e^{-h*(\omega_{i}-0.5)}\right)}-\gamma_{i}x_{i} \end{array}$$ (1)

The parameter *h* determines the form of the curve; when *h* is very close to 0, the curve becomes a straight line, while with values close to 100, the curve approximates a step function. The parameter *ω*_*i*_ is the continuous form of *F*_*i*_(*X*_*i*_1__(*t*), *X*_*i*_2__(*t*), …, *X*_*i*_*k*__(*t*)) used in the Boolean model, and *γ*_*i*_ is its degradation rate. Detailed information about the continuous model can be found in S2 Text.

## Results

### The regulatory network recovers a dynamical model of *A. thaliana* CC

**Fig 1:**
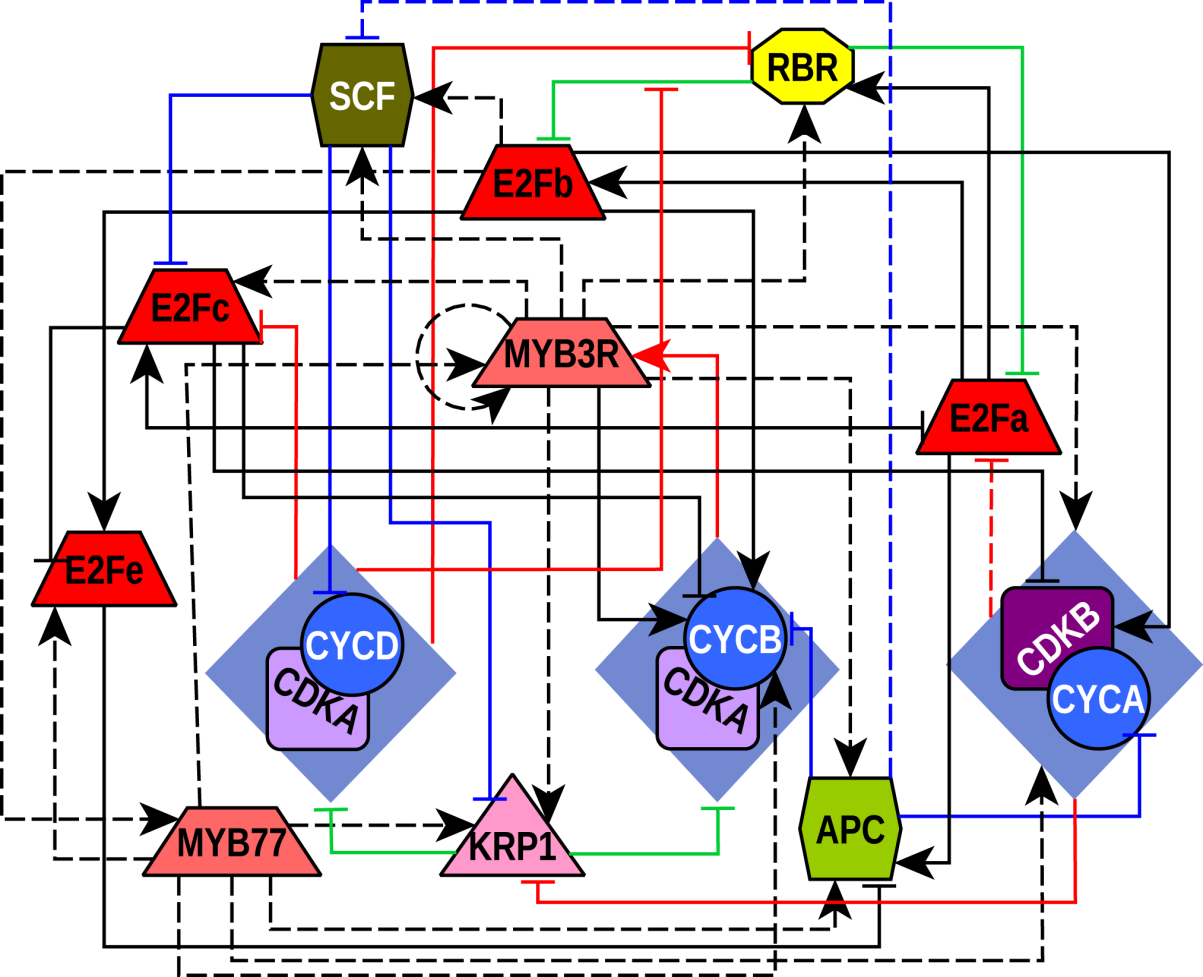
Regulatory network of the *A. thaliana* CC. The network topology depicts the proteins included in the model as well as the relationship among them. Nodes are proteins or complexes of proteins and edges stand for the existing types of relationships among nodes. The trapezoid nodes are transcription factors, the circles are cyclins, the squares are CDKs, the triangle represent stoichiometric CDK inhibitor, the hexagons are E3-ubiquitin ligase complexes and the octagon is a negative regulator of E2F proteins. Edges with arrow heads are positive regulations and edges with flat ends illustrate negative regulations. The red edges indicate regulation by phosphorylation while blue ones indicate ubiquitination, the green ones show physical protein-protein interactions and the black edges transcriptional regulation. Only CDK-cyclin interactions are not represented with a line. Interactions to or from rhombuses stand for interactions that involve the CDK as well as the cyclin. A solid line indicates that there is experimental evidence to support such interaction and dotted lines represent proposed interactions grounded on evidence from other organisms or *in silico* analysis.

The CC model proposed here integrates and synthesizes published data for *A. thaliana* CC components interactions, as well as some molecular data from other organisms (mammal and yeast), that we propose as predictions for *A. thaliana* CC regulation, and assume to be conserved among all eukaryotes. The whole set of interactions and nodes included in the model and detailed in Tables 1 and 2 are shown in Fig 1. Four types of molecular interactions can be distinguished: (i) transcriptional regulation, (ii) ubiquitination, (iii) phosphorylation and (iv) physical protein-protein interactions. Additionally, an *in silico* analysis of transcription factors and promoters was carried out, in order to further substantiate 16 predicted interactions in the GRN (these are: E2Fb → MYB77; MYB77 → E2Fe, MYB3R1/4, KRP1, CYCB1;1, CYCA2;3, CDKB1;1 and CCS52A2; MYB3R1/4 → SCF, RBR, CDKB1;1, CYCA2;3, APC/C, KRP1, E2Fc and MYB3R1/4). The logical rules are available in S1 Text.

**Fig 2:**
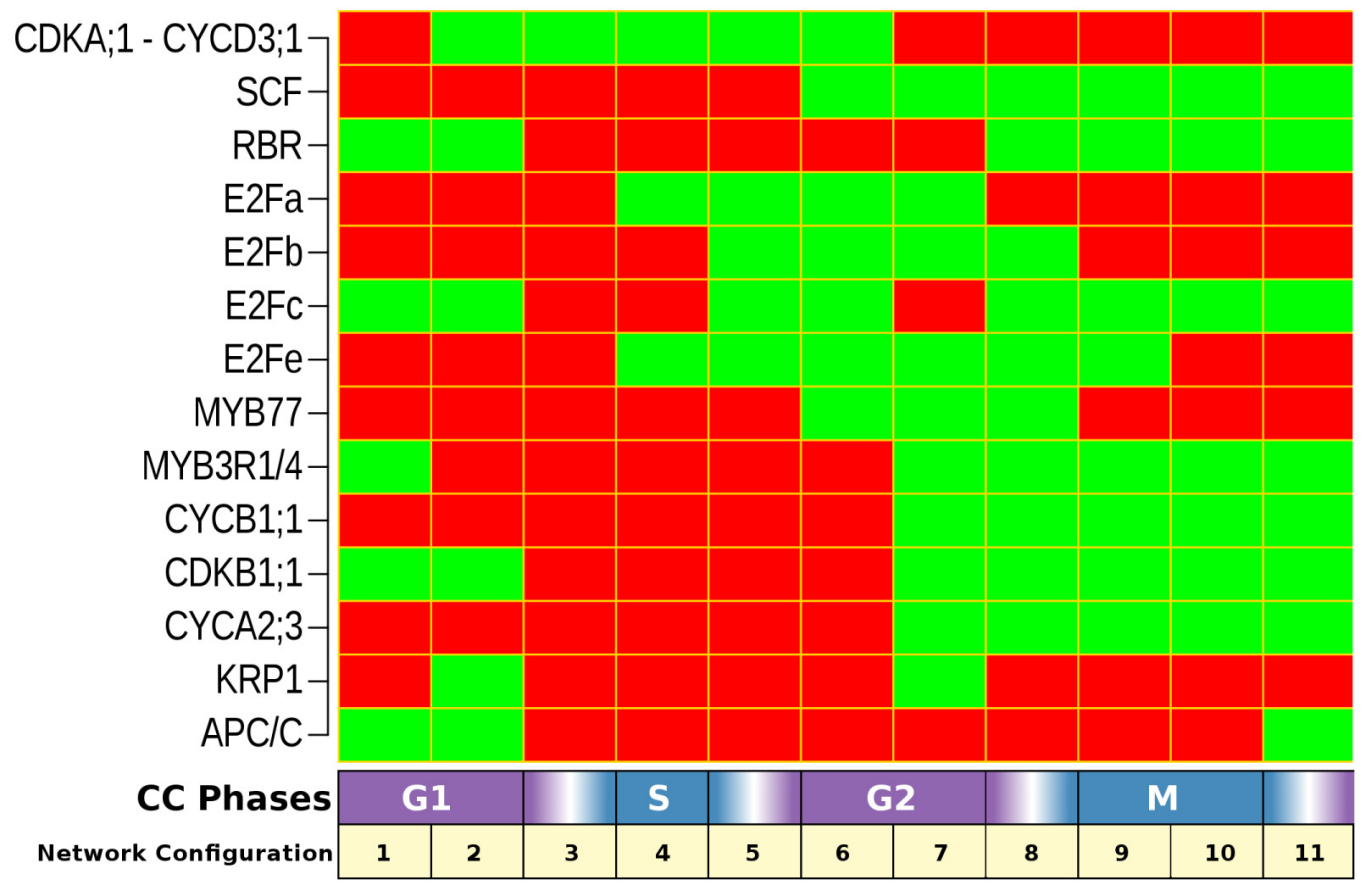
Attractor corresponding to a dynamic network of CC in *A. thaliana*. 100% of the whole set of network configurations converges to a unique attractor composed by 11 configurations. Each column is a network configuration (state of each network component) and the rows represent the state of each node during CC progression. The squares in green indicate components that are in an “ON” state and the ones in red are nodes in an “OFF” state.

Our results show that the nodes and interactions considered are sufficient to recover a single robust cyclic steady state, and thus the cyclic behavior of the components considered. Such behavior closely resembles the periodic patterns observed during actual CC progression, Fig 2. The first two columns or network configurations match a G1 state, given that during the early G1 phase, the CDKA;1-CYCD3;1 complex is absent or inactive by the presence of KRP1 [92, 93, 108]. The CDKA;1-CYCD3;1 state is given only by the presence of CYCD3;1 since CDKA;1 is always expressed in proliferative cells [68]. To facilitate understanding, in Fig 2 the complex CDKA;1-CYCD3;1 is shown instead of only CYCD3;1. The absence of mitotic cyclins (CYCA2;3 and CYCB1;1) at this stage [28, 38], as well as the APC/C presence until the early G1 phase, which is needed for the mitosis exit, also coincides with experimental observations [44, 109, 110]. The presence of the RBR protein in G1-phase implies an inactive state of the E2F, as expected [33, 111, 112]. Then, the third column resembles G1/S transition, where the presence of CDKA;1-CYCD3;1 complex would be inducing RBR phosphorylation and its inactivation [32]. In the fourth configuration, the S-phase is represented by RBR inactivation and E2Fa/b transcriptional activation [113]. In the fifth and sixth configuration, E2Fc state returns to “ON” but RBR state is kept in “OFF”, which indicates that transcription driven by E2Fa and E2Fb can still happen. Indeed, the E2Fb factor appears from the fifth configuration and it is consistent with their function regulating the expression of genes needed to achieve the G2/M transition. In the sixth configuration, MYB77 is turned on, although in synchronization experiments it has been observed to be on until the beginning of mitosis [73]. During G2-phase the MYB transcription factors and KRP1 are expressed [31, 73, 93], the former would maintain dimers of CDKA;1 and mitotic cyclins inactive; and together, this data is consistent with what is observed in the seventh configuration of the CC attractor. In the eighth column, KRP1 is lost because it was phosphorylated by CDKB1;1-CYCA2;3, which is active in the G2/M transition and the onset of mitosis [27]. The phosphorylation of KRP1 drives its degradation and posterior activation of mitotic complexes such as CDKA;1-CYCB1;1 to trigger mitosis [21, 78] (configuration 9 and 10 in Fig 2). The lack of APC/C at the onset of mitosis is determinant for the accumulation of the mitotic cyclins, but APC/C presence is necessary for the mitosis exit [110], which occurs in the eleventh configuration of the attractor (Fig 2). Thus, our CC GRN model recovers a unique attractor of eleven network configurations (Fig 2), which shows a congruent cyclic behavior of its components with that observed experimentally. This result validates that the proposed set of restrictions converge to a single cyclic behavior, which is independent of the initial conditions. A further validation of the proposed CC model, would imply that the recovered cyclic attractor is robust to permanent alterations, as is the case for real CC behavior that is highly robust to external and internal perturbations [14, 58, 114, 115].

### The CC Boolean model is robust to alterations

To provide further validation for the proposed CC regulatory network, we performed robustness analyses of the attractor to four types of alterations in the logical functions of the model. First, we altered the output of each logical rule by systematically flipping one by one, each one of their bits. We found that 87.47% of the perturbed networks recovered the original attractor, while 1.77% of the altered networks maintained the original attractor and produced new ones (see supplementary material S3 Text for details). In contrast, the remaining 10.76% of alterations reduced the number of network configurations of the original attractor. In the second robustness analysis, after calculating the transitions between one network configuration to the next one, one bit (i.e. the state of a node) of this next configuration is randomly chosen and its value changed. Then, the network is reconstructed and its attractors recovered again. This procedure was repeated 100 times, thus we found that in 88.2 ± 3.2 out of the 100 perturbations (mean ± SD) the original attractor was reached. These results suggest that the proposed GRN for *A. thaliana* CC is robust to alterations as expected and in coincidence with previous GRN models proposed for other developmental processes [116, 117].

**Fig 3:**
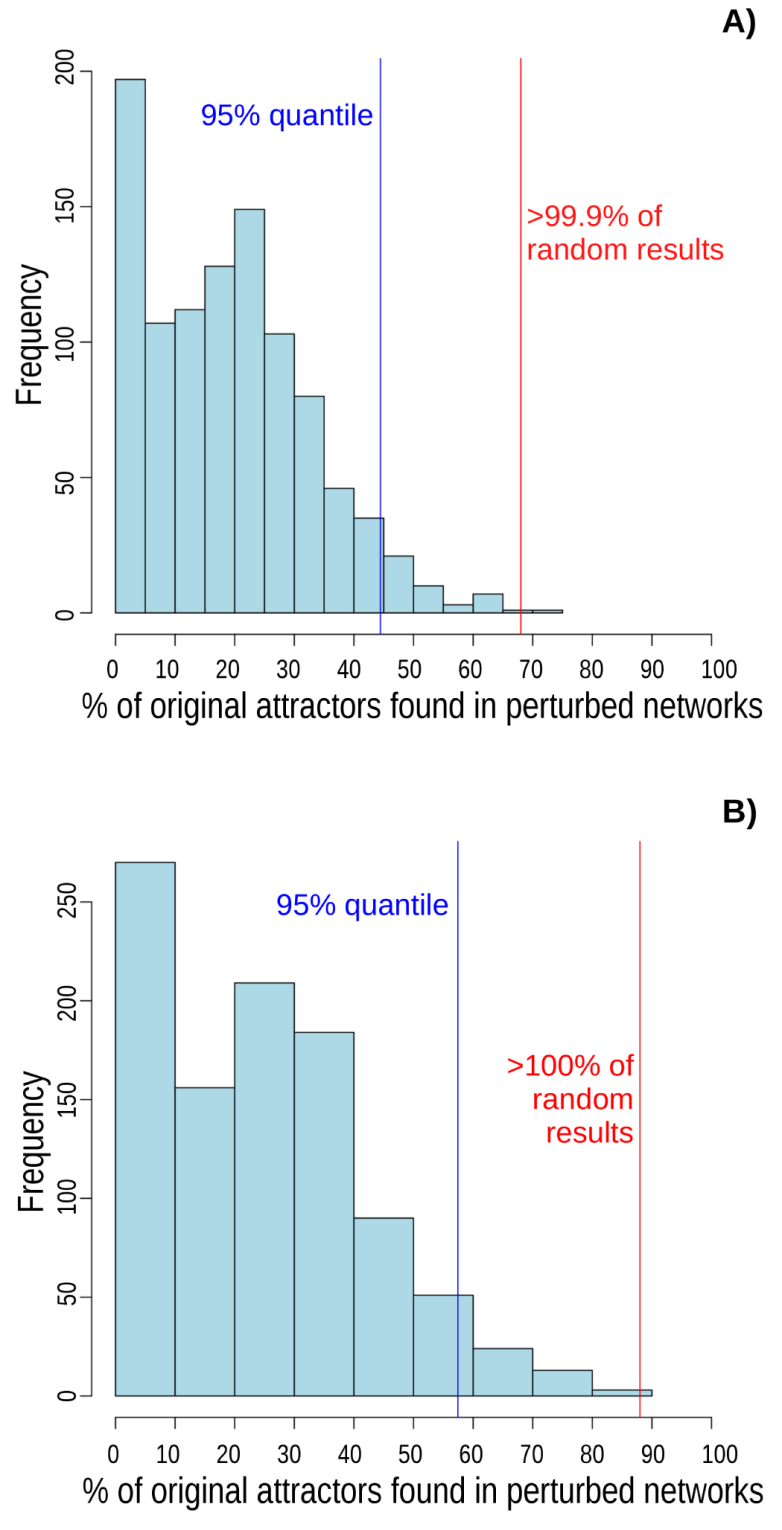
Attractor robustness analysis. Random networks with similar structure to *A. thaliana* CC GRN were less tolerant to perturbations than original CC GRN. The frequency of perturbations that recovered the original attractor after a perturbation in the Boolean functions, is shown in: (A), where the red line indicates that *A. thaliana* CC GRN recovers its original attractor in 68% of perturbations (the median of random networks was 18.55% and mean 19.12% ± 13.86 SD). When transitions between network configurations are perturbed (B), *A. thaliana* CC GRN recovers its original attractor in 88% (vertical red line) of perturbations, while the median of random networks that recover the original attractor was 24.2% (mean 24.6% ± 18.2 SD). Vertical blue line indicates the 95% quantile. 1000 random networks were analyzed.

To confirm that the robustness recovered in these two types of analyses is a specific property of the network under study, we performed robustness analyses of randomly generated networks with similar structures (same number of input interactors for the logical functions) to the one proposed here for the *A. thaliana* CC regulatory network, and compared the above robustness analyses results to those recovered for equivalent analyses for the random networks. We generated 1000 random networks. Then, 100 copies of the random and of our network were done. In each copy we randomly flipped the value of one bit in one logical function (to confirm the first robustness analysis), or in one next configuration (for the second robustness analysis). When perturbations are made in logical functions, the *A. thaliana* CC GRN recovers its attractor in 68% of perturbations, while the median of percentage of cases in which such attractor was recovered in the random networks was only 18.55% (mean 19.12%±13.86 SD, Fig 3A). The difference between the 68% of this latter analysis and the 87.47% of the first robustness analysis could be due to sampling error. If transitions between network configurations are perturbed, the median of original attractors recovered in random networks is 24.2% (mean 24.6% ± 18.2 SD). In contrast, the original attractor of *A. thaliana* CC GRN was found in 88% of perturbed networks starting with that grounded on experimental data (Fig 3B). These results confirm that the CC GRN proposed here is much more robust than randomly generated networks with similar topologies and suggests that its robustness is not due to overall structural properties of the network.

**Fig 4:**
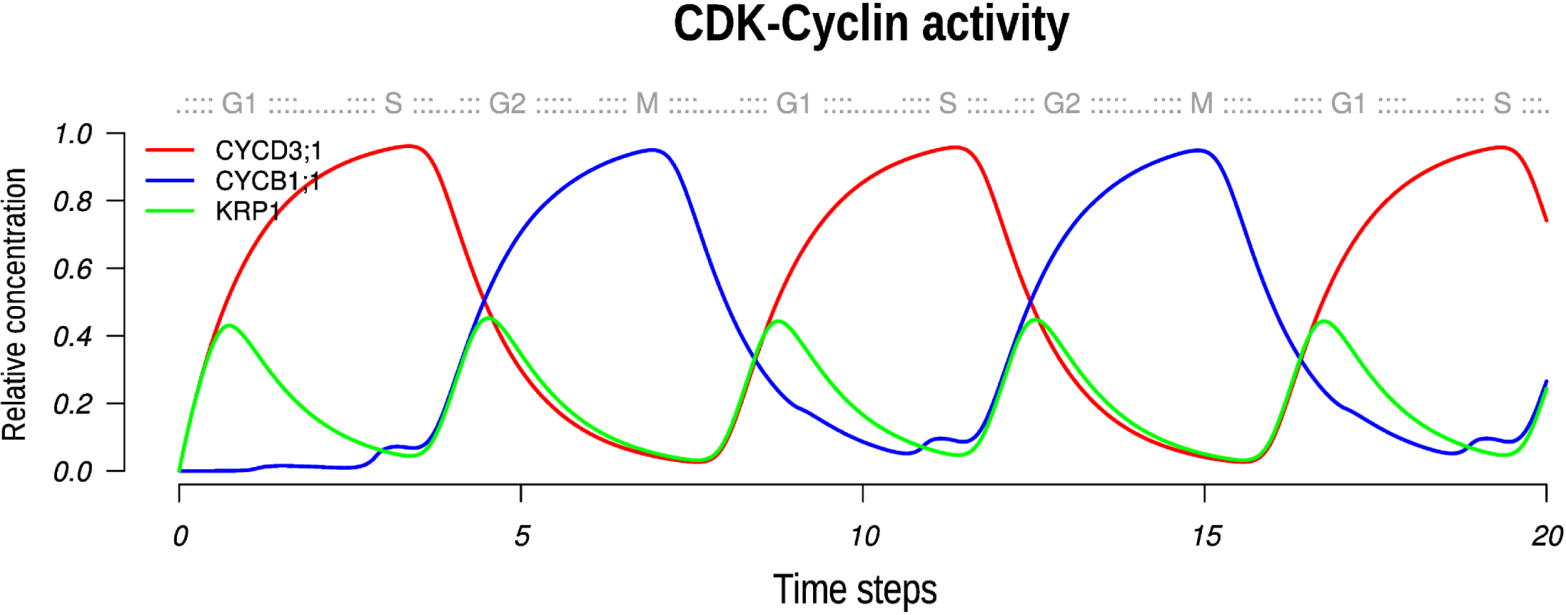
Continuous version of the *A. thaliana* CC Boolean model. In this graph we show the activity of the CDKA;1-CYCD3;1 and the CDKA;1-CYCB1;1 complexes as a function of the amount of cyclins, and KRP1 inhibitor. The CDK-cyclin activity is the limiting factor to pass the G1/S and the G2/M checkpoints. A little more than two complete CC are shown (upper horizontal axis) to confirm that oscillations are maintained.

Boolean models can produce cyclic dynamics as an artifact due to their discrete nature and the time delays implied. To address this issue we approximated the Boolean model to a continuous system of differential equations following [106, 107, 118, 119]. To recover steady states of such continuous system, the continuous versions of the GRN were evaluated for 1000 different randomly picked initial conditions (See S2 Text). In all cases and independently of the methodology (i.e. [106, 107] or [118, 119]), we recovered the same limit cycle steady state. In the continuous model, key cyclins for the main phase transitions, CYCD3;1 and CYCB1;1, have an oscillatory behavior that is not attenuated with time (Fig 4). Importantly, this result is robust to changes in the decay rates or alterations of the *h* parameter that affects the shape of activation function (see details in S2 Text); the limit cycle was recovered in 92.86% of the cases. The results of the continuous model corroborate that the limit cycle attractor recovered by the Boolean version, is not due to an artifact associated to the discrete and synchronous nature of the Boolean model, but is rather an emergent property of the underlying network architecture and topology. In addition, the recovery of the cyclic behavior of the continuous model constitutes a further robustness test for the Boolean model.

Previous studies have also tested asynchronous updating schemes [46]. In this study we have used a continuous form of the model to discard that the recovered cyclic attractor is due to an artifact owing to the discrete and synchronous nature of the model used. Future studies could approach analyses of asynchronous behavior of the model by devising some priority classes distinguishing fast and slow processes, and thus refining the asynchronous attractor, under a plausible updating scheme. On the other hand, biological time delays may be involved in CC progression, but they are not enough for irreversibility. The CC unidirectionality has been proposed to be a consequence of system-level regulation [120], here we hypothesize that the ordered transitions of *A. thaliana* CC are an emergent property of network architecture and dynamics.

### Simulated loss- and gain-of-function mutants recover observed patterns: normal CC and endocycle

**Fig 5:**
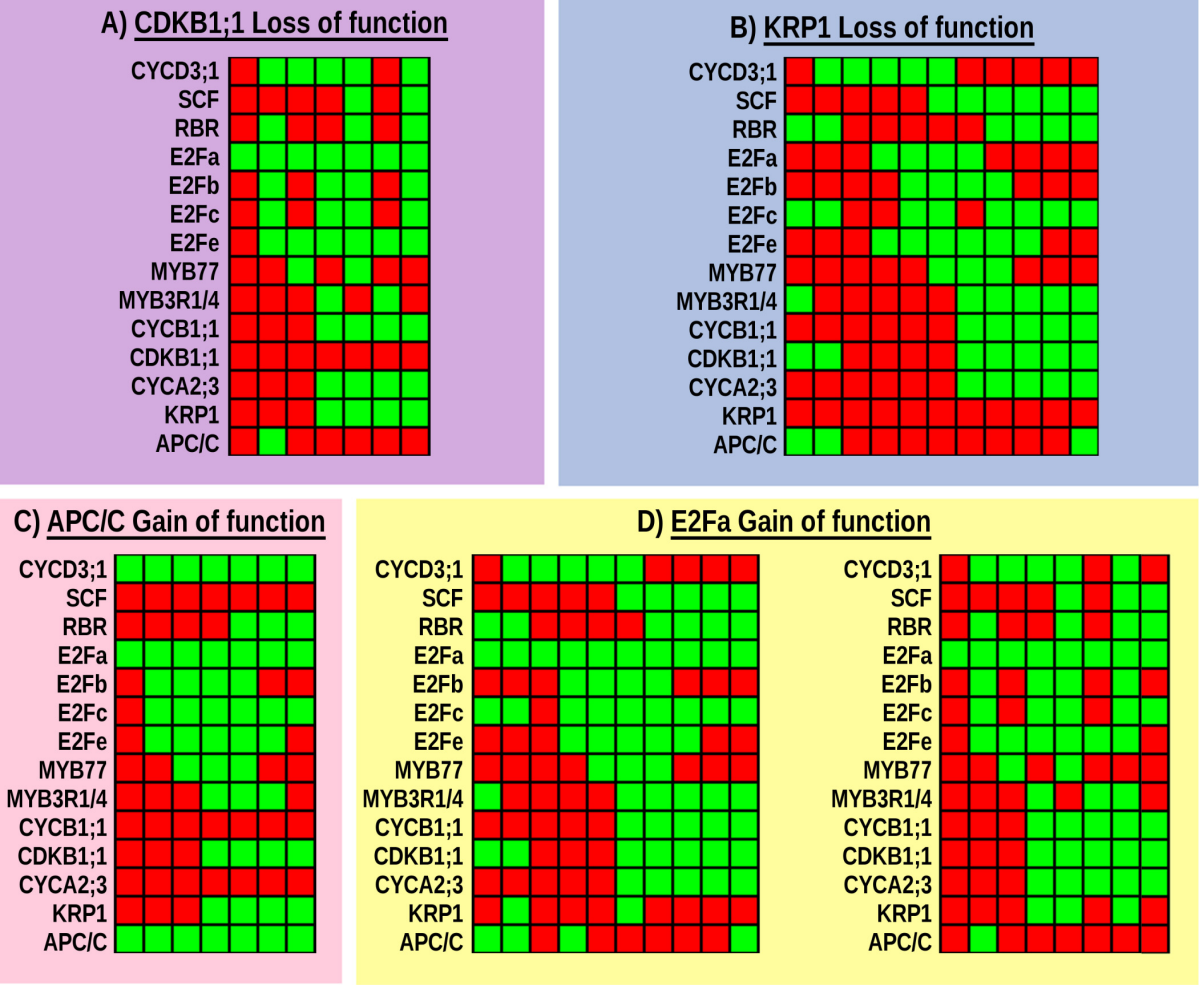
Attractors recovered by simulations of loss- or gain-of-function mutants of four CC components. (A) The simulation of loss of CDKB1;1 function produced only one cyclic attractor with period 7 that resembles G1 → S → G2 → G1 cycle, whereas in (B) with simulation of loss of KRP1 function, one cyclic attractor was attained, which has period 11 and comprises 100% of the initial conditions. This attractor is almost identical to WT phenotype but without KRP1. With the simulation of APC/C gain-of-function, a single attractor with period 7 was recovered, which is shown in (C) and is consistent with an endoreduplication cycle. Attractors obtained with the simulation of E2Fa overexpression are shown in (D). Two attractors were found, one of them has period 10 and the 40.48% of the initial conditions converge to that cycle that is closely similar to the WT CC attractor. The second attractor that correspond to E2Fa overexpression has period 8 and it is very similar to the endoreduplication attractor of loss of CDKB1;1 function, which comprises 59.52% of possible network configurations.

An additional validation analysis for the proposed *A. thaliana* CC model implies simulating loss- and gain-of-function mutations and comparing the recovered attractors with the expression profiles documented experimentally for each mutant tested. We simulated mutants by fixing the corresponding node to 0 or 1 in loss- and gain-of-functions mutations, respectively. The recovered altered configurations are summarized in S4 Text, and in Table 3 as well as in Table 4 for gain- and loss-of-function mutants, respectively. The simulated mutant attractors are coherent with experimental data in most cases [2, 21, 23, 30, 35, 37, 43, 44, 76, 79, 80, 88, 90–93, 103, 108, 109, 111, 113, 114, 121–129]. In Fig 5 we show a representative example of attractors recovered by simulations of CDKB1;1 and KRP1 loss-of-function and APC/C and E2Fa gain-of-function mutants. It is noteworthy that several simulated mutants, such as mitotic cyclins or B-type CDK loss-of-function, converge to a cyclic attractor that corresponds to the configuration observed under an endoreduplicative cycle (e.g. Fig 5A). In such attractors, endoreduplication inductors, such as APC/C, KRP1 and E2Fc [37, 78, 130] are present, at least in some network configurations (Fig 5A, 5C and 5D-right). Another outstanding feature of these mutant attractors is that, although mitotic CDK-cyclin complex may be present, it is inhibited by KRP1, therefore there is no CDK-cyclin activity to trigger the onset of mitosis. These data are coincident with the reported regulation during the onset of endoreduplication [21]. In the attractors where E2Fa coincides with alternating states of RBR, it suggests that DNA replication may occur (Fig 5). Likely due to plant redundancy, some mutations do not produce an obvious impaired phenotype. Such is the case of KRP1 loss-of-function, in which loss-of-function simulation, a cyclic attractor identical to the original one is recovered, as is expected (see Table 4), because such mutants do not show an evident altered CC behavior (Fig 5B) [93].

**Table 3:** Phenotypes of gain-of-function mutations in CC components.

Node	Phenotypes of gain of function	Recovered attractor(s)	Refs.	Model
**CYCD3;1**	Inhibition of CC exit, increases division zones and ectopic divisions. Decreases G1 phase duration and increases G2 duration. Delays expression of G2/M genes.	Fixed-point attractor of G2-phase.	[108, 121]	PA
**SCF**	SKP2A gain-of-function enhances proliferation, and increases number of cells in G2/M and ploidy levels decrease.	Oscillates between G1 and S.	[122, 123]	NR
**RBR**	CC arrest, cells in root apical meristem lose CYCB1;1 expression; in rice, the number of cells synthesizing DNA decrease.	Fixed-point attractor characterizing G1 arrest.	[2, 88]	A
**E2Fa**	Mitosis and endoreduplication are promoted.	One attractor comprising 40.48% of initial conditions that is a WT CC. The other closely resembles an endocycle but APC/C activity is lower (59.52% of configurations).	[111, 113]	A
**E2Fb**	Cell division is induced but endoreduplication is suppresed; CC duration and cells are shorter, and the amount of S-phase transcripts increases.	Similar to WT but with a shorter S phase.	[79, 80]	A
**E2Fc**	Overexpression of a non-degradable form of E2Fc leads to larger cells or a lack of division.	Fixed-point attractor where only E2Fc and CYCD3;1 are present, congruent with a CC-arrest.	[35]	PA
**E2Fe**	Reduces ploidy levels.	CC arrest in M phase.	[44]	PA
**MYB77**	Plants are stunted but there is no information about how CC could be affected.	CC arrest in a mitotic state.	[76]	-
**MYB3R1/4**	No available data about how it could alter cell division.	Two fixed-point attractors of CC arrest at early G1 phase, state of E2Fa varies among them.	-	-
**CYCB1;1**	Root growth enhanced, slightly small cells.	WT CC	[124]	A
**CDKB1;1**	Does not seem to affect CC behavior.	WT CC	[125]	A
**CYCA2;3**	Not enough to produce multicellular trichomes but the proportion of polyploid cells is less.	WT CC	[103]	A
**KRP1**	CC arrest and inhibition of cell proliferation, G2 phase is longer; a weak overexpression of KRP2 led to an increment in DNA ploidy.	Attractor with period 2 oscillating between G1 and G1/S transition.	[21, 30, 126]	PA
**APC/C**	Gain-of-function of APC/C subunit CCS52A2 enhanced endoreduplication entry; more cells with increased DNA ploidy.	Cyclic attractor pointing to endocycle.	[44]	A

Summary of mutant phenotypes and recovered attractors simulating that mutation. **A** means that the result of simulation is in Agreement with the data available in the reference(s). **PA** means it is Partially Agrees with evidence, because not all expected traits were reproduced by the attractor but this does not contradict the mutant phenotype. **NR** are attractors that do not make sense with expected behavior and therefore, the model did Not Recover the mutant phenotype.

**Table 4:** Phenotypes of loss-of-function mutations in CC components.

Node	Phenotypes of loss of function	Recovered attractor(s)	Refs.	Model
**CYCD3;1**	When this cyclin is depleted by sucrose starvation, cells are arrested in G1 phase; in adult leaves, triple mutant of *cycd3;1–3* led to a diminished number of cells.	Period 2 oscillating between G1 and G1/S transition.	[23, 92]	A
**SCF**	Plants with a diminished level of SKP2 do not show obvious affected development but KRP1 is accumulated.	Similar to a normal CC but endoreduplication would be favored by the KRP1 stabilization.	[93]	A
**RBR**	Proliferation is promoted and cell differentiation is impaired; downregulation of RBR in rice promotes an increase of cells in S-phase.	One attractor of a normal CC (includes 81.98% of possible configurations) and other attractor oscillates among G2-S-G2 (18.02% of configurations).	[127]	A
**E2Fa**	Expression of E2Fb, RBR and other CC regulators decrease; more cells in G1 and G2 with respect to WT.	Fixed-point attractor with E2Fe and CYCD3;1 present suggesting an arrest in a Gap phase.	[90]	PA
**E2Fb**	Without information.	Fixed-point attractor representing the G1/S transition.	-	-
**E2Fc**	Mitotic proteins such as CYCB1;1 have increased expression, ploidy is reduced.	Fixed-point attractor of M phase arrest.	[37, 91]	PA
**E2Fe**	Increased endoreduplication.	Attractor of endoreduplication (period 7).	[44]	A
**MYB77**	Lower density of lateral roots, inconclusive data to evaluate simulation.	CC of seven configurations.	[76]	-
**MYB3R1/4**	Lower levels of G2/M transcripts, incomplete cell division, some embryos only have one cell with multiple nuclei.	2 attractors, the first seems a three-configurations endocycle, and the second is a CC of seven configurations where APC/C is always absent.	[43]	A
**CYCB1;1**	Cyclin widely used as a marker of cell proliferation, its absence is associated with differentiated cells.	Attractor characterizing endocycle (period 8), intriguingly APC/C is never present.	[128]	-
**CDKB1;1**	Overexpression of a dominant negative allele leads to enhanced endoreduplication.	Attractor of endoreduplication (period 11).	[129]	A
**CYCA2;3**	In null mutants, cells with 2C DNA content decreases before than in WT, endocycles begin before and are faster than in WT.	Attractor which is an endocycle (period 7).	[103]	PA
**KRP1**	No evident phenotypic effects observed but relative kinase activity increases to 1.5 in relation to WT.	A CC without alterations.	[114]	A
**APC/C**	Loss of CCS52A2 function (activator subunit of APC/C) produces a decrement in the number of meristematic cells without affecting endoreduplication index; cells in quiescent center become mitotically active.	Fixed-point attractor of a CC arrest previous to conclude mitosis.	[109]	PA

Summary of mutant phenotypes and recovered attractor when that mutation was simulated. Abbreviations in Model column are as in Table 3.

Interestingly, the simulation of a constitutively active APC/C also converges to a single cyclic attractor, which corresponds to an endoreduplication cycle, since it has Gap and S phases, but lacks an M-phase configuration. This coincides with the experimental observation that the overexpression of one of the APC/C subunits (CCS52A) promotes entry to an endocycle [44] (see Table 3). Another interesting example is the gain-of-function mutation of E2Fa that yields two cyclic attractors, one corresponding to the normal CC cycle and the other one to an endocycle (Table 3). It has been shown that this gene is required for both processes [111] that are apparently exclusive, although in both processes the DNA replication occurs and among E2Fa targets there are genes required for S-phase. Thus our model suggests that the regulation of E2Fa at the end of G2 phase is decisive for CC exit and transition to endoreduplication. In this E2Fa gain-of-function simulation, we found an inconsistency with APC/C because this E3 ubiquitin ligase is decisive for endoreduplication, while in the simulated attractor is only present in one network configuration (Fig 5D-right). Such behavior observed in the endoreduplication attractor for E2Fa gain-of-function leads to unstable activity in the CDK-cyclin complex (Fig 5D), thus suggesting that the increase in APC/C is required for endoreduplication entry as well as its progression. In the attractor of the simulated APC/C gain-of-function, the states of the CYCD3;1, SCF, E2Fb, E2Fc and MYB nodes are more stable than in endoreduplication attractors of CDKB1;1 loss-of-function or E2Fa gain-of-function, where E2Fb, E2Fc and MYB factors expression states alternate between “ON” and “OFF” (Fig 5).

We highlight APC/C gain-of-function simulations, as it provides a possible mechanism for plant hormones action over the CC machinery and, thus how such key morphogens regulate cell proliferation patterns. Recently, Takahashi and collaborators reported a direct connection between cytokinins and CC machinery in *A. thaliana* root [131]. The authors showed that ARR2, a transcriptional factor of cytokinins signaling, induces expression of APC/C activator protein CCS52A1. Our simulated APC/C gain-of-function is congruent with that observation, since it reproduces the configuration attained by a cell entering an endocycle when APC/C activity is enhanced (Fig 5C), as it happens at the elongation zone of *A. thaliana* root. Therefore, our model is able to recover the attractors of loss- and gain-of-function mutant phenotypes reported experimentally, and it thus provides a mechanistic explanation for observed patterns of expression in both normal CC and during endoreduplication cycles or endocycle.

### Plant E2Fc and KRP1: validation of *A. thaliana* CC GRN

**Fig 6:**
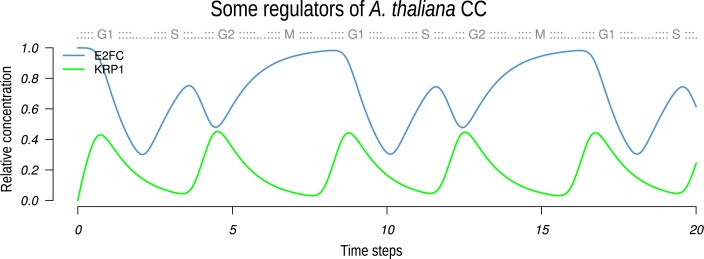
Dynamical behavior of E2Fc and KRP1 according to the continuous model. These nodes were chosen by their peculiar pattern of expression, which was qualitatively recovered by the Boolean and continuous models.

We test if the CC GRN recovers the periodic patterns observed in synchronization experiments of *A. thaliana* CC molecular components. Interestingly, the E2Fc repressor and KRP1 are regulators that have two short lapses of expression in the attractor recovered in the continuous model (Fig 6), and experimentally they also show two peaks of expression when synchronized with aphidicolin [74]. In such synchronization experiments, the expression of E2Fc increases from late S to middle G2, but then it decreases dramatically in late G2. In the model, E2Fc appears from S to G2 phase, and then a second increment of E2Fc expression in G2/M is observed. The latter correspondence is a further validation of the CC GRN model proposed here. Furthermore, synchronization experiments using sucrose have shown that KRP1 is expressed previous to G1/S transition and before mitosis [132], in a similar way that occurs in the model. More recently it has been proposed that KRP1 has a role during G1/S and G2/M transitions [93]; the latter should be important for endoreduplication control [78]. Once again, such roles and expression profiles are consistent with the recovered active state of KRP1 in our model.

In contrast with the consistent behaviors of E2Fc and KRP1 components to recovered results with our model, E2Fe results do not coincide with previous observations. In our model this E2F factor presents only one peak from S to early M phase, but according to synchronization experiments [69], E2Fe has two peaks of expression. One of its peaks is due to regulation by other E2F family factors during S phase, while the G2/M peak could be due to MSA elements. Indeed, when the regulatory motifs for E2F binding are deleted from E2Fe, it can still be expressed although at lower levels [96], suggesting that additional transcription factors regulate its expression. Such factors could belong to the MYB family as suggested for the *A. thaliana* CC GRN proposed here.

## Discussion

The canonical cyclic behavior of eukaryotic cells as they go from DNA duplication to cytokinesis suggests that a conserved underlying mechanism with shared molecular components and/or regulatory logic should exist. While yeast and animal CC have been thoroughly studied and modelled, plant CC is less studied and no comprehensive model for it has been proposed.

In this study we put forward a Boolean model of the *A. thaliana* CC GRN. We show that this model robustly recovers a single cyclic attractor or steady state with 11 network configurations. Such configurations correspond to those observed experimentally for the CC components included here at each one of the CC stages. In addition, the canonical order of sequential transitions that is recovered also mimics the observed temporal pattern of transition from one configuration to another one along the CC (Fig 2). The fact that the 16,384 initial conditions of the proposed system converge to this single cyclic attractor already suggests that the GRN comprises a robust module that integrates the necessary and sufficient set of components and interactions to recover molecular oscillations experimentally observed. The proposed GRN is also robust to alterations, being similarly robust to previously published models for other cell differentiation or developmental modules [116, 117, 133]. The model is validated because it recovers *A. thaliana* wild type and altered (in gain- and loss-of-function) configurations and cycling behaviors. The comparison between experimentally observed and recovered gene configurations is summarized in Tables 3 and 4.

Some cyclins such as CYCD3;1 and CYCB1;1, important components during G1/S and G2/M transitions, show a mutually exclusive regulation, as occurs in a predator-prey Lotka-Volterra dynamical system [134], even though they do not interact directly. Their mutual exclusion is achieved thanks to the coordinated expression of genes with specific proteolytic degradation capacity. Our cyclic attractor shows two transcriptional periods, one of them in S-phase regulated by E2F-RBR pathway, and the second one operating at a time previous to M-phase and regulated by MYB transcription factors. The SCF and APC/C ubiquitin ligases work during G2-to-M phases, and during mitosis exit, respectively. Therefore, the fourteen nodes and their interactions proposed in the CC GRN constitute a necessary and sufficient set of restrictions to recover the oscillations of node states characteristic of CC phases.

Two alternative possibilities could drive CC progression in actual organisms. The first would imply that transitions from one CC state to the next would require external cues, like the cell size. The alternative possibility is that CC progression and the temporal pattern of transitions among stages are both emergent consequences of an underlying complex regulatory network, and do not require external cues, or these only reinforce such temporal progression emergent from complex underlying regulatory interactions. Our CC GRN model supports the latter. This does not imply that several internal or external signals or molecules, such as hormones or other types of cues could alter the CC. Therefore, the two alternative possibilities are not exclusive but they likely complement or enhance each other. Indeed, *A. thaliana* CC is regulated by plant hormones, light, sucrose, osmotic stress [135] or oxidative stress [136]. These could now be modelled as CC modulators.

In the model proposed here we avoided redundancy. For instance, the KRP1 node represents the KRP family members that share several functions. Also the metaphase-anaphase transition could be added to the model when more data about APC/C regulation (i.e. negative feedback loop comprising CDK-cyclin complexes, or the regulation of Cdc20 homologues) becomes available in plants. Apparently, these simplifications did not disrupt the main features of the *A. thaliana* CC, since the cyclic behavior distinctive of the CC components was correctly recovered.

### A mechanistic model for the *A. thaliana* CC: novel predictions

Our proposed GRN model suggests some predictions regarding the regulation of certain CC components in *A. thaliana*. Such predictions can be classified into two types. The first type pertains to those recovered by *in silico* promoter analysis. The predictions of the second type were inferred from data of other eukaryotes, because they seem to imply conserved components and some evidence from *A. thaliana* suggested that these interactions are part of the CC GRN in *A. thaliana*. Three interactions belong to the second type, E2Fb → SCF, CDKB1;1-CYCA2;3 ⊣ E2Fa and APC/C ⊣ SCF (see Table 1 for a synthesis of hypothetical interactions). Although some evidence supports the idea that these interactions could exist in *A. thaliana*, they should be corroborated with additional experimental examination.

Our model provides a dynamic explanation to the cyclic behavior of certain transcription factors and predicts a novel interaction for E2F and MYB regulators; they connect waves of periodic expression that seem to be key for the robust limit cycle attractor that characterizes CC behavior. Interestingly, previous studies have shown that such periodic transcription can be maintained even in the absence of S-phase and mitotic cyclins [4], which underpin the role of a transcription factor network oscillator for the correct CC progression [137]. A regulatory interaction between E2F and MYB factors (or among the equivalent regulators) may be conserved among other eukaryotes (e.g. mammals and yeast), but there is no experimental support yet for it in *A. thaliana*. After looking for the same direct evidence in *A. thaliana* and not finding it, we thought about an alternative regulatory mechanism that consists in transcription factors acting between E2F and MYB. Hence, we decided to analyze the important transcription factor families known so far, to find out if one of their members could be mediating the regulation between E2F and MYB. The TCP (for Teosinte branched 1, Cycloidea, PCF) and the MYB family were chosen because they have been reported to be involved in CC regulation [42]. Based on their gene expression patterns and promoter sequence analysis, MYB77 was our best candidate: it is expressed at the beginning of M phase, and could be regulated by E2F and regulator of MYB (see Table 1). A second possibility might be that several tissue-specific transcription factors are involved in E2F-MYB genetic regulation (e.g. GL3, MYB88, SHR/SCR [17], MYB59 [138] or even members of the MADS box gene family could be implied). Indeed, we have recently documented that a MADS-box gene, XAL1, encodes a transcription factor that regulates several CC components (García-Cruz et al., in preparation).

### *A. thaliana* CC in comparison to animal and yeast CC

Differences among eukaryotic CCs allow us to recognize or characterize alternative mechanisms for the regulation of CC. The first difference between GRN of *A. thaliana* CC and that of other eukaryotes, concerns the number of duplicates of some key regulators. *A. thaliana* has up to ten copies of some of the genes that encode for CC regulators (e.g. families of cyclins or CDK), while yeast, mammals or the algae *Ostreococcus tauri*, have much fewer duplicates [20, 139–141]. The only exception concerns the homologues of Retinoblastoma protein, of which there are three members in humans and mouse, and only one copy in *A. thaliana* [127]. Future models should address the explicit role of CC duplicated components in the plastic response of plant development to environmental conditions. Being sessile, such developmental adjustments, as plants grow under varying environments, are expected to be more important, complex and dynamic than in motile yeast and animals. One possibility is that different members of the same gene family are linked to different transduction pathways of signals that modulate CC dynamics.

The second difference among *A. thaliana* and other CC was regarding the transcriptional regulation throughout the GRN underlying it. For instance, *S. cerevisiae* does not have RBR or E2F homologues, but instead has Whi5, Swi4,6 and Mbp1 proteins which perform equivalent regulatory functions to the former CC components [142, 143]. *S. cerevisiae* does not have any MYB transcription factors but it presents other transcriptional regulators, such as Fkh1/2, Ndd1 and Mcm1 [142, 144, 145], which regulate the G2/M transition in a similar way to MYBs in mammals.

Contrary to the conservation in G1/S transition [15, 67], molecular components controlling G2/M transition seem to vary among different eukaryotes. It seems that molecules such as WEE1 kinase and CDC25 phosphatase are not conserved. In *A. thaliana*, CDC25-like has phosphatase and arsenate-reductase functions [146], while *A. thaliana* WEE1 phosphorylates monomeric CDKA;1 *in vitro* [147], and *Nicotiana tabacum* WEE1 inhibits CDK activity *in vitro* [148]. However the lack of any obvious mutant phenotype of CDC25 or WEE1 loss-of-function mutants predicts that these genes are not involved in the regulation of a normal CC. Additionally, although WEE1 has a role during DNA damage [146, 149], does not seem to have a CDKA;1 recognition domain [150]. CDC25-like does not have the required sites for CDKA;1 recognition [150]. In summary, the positive regulatory feedback between CDKA;1 and CDC25-like, as well as the mutual-inhibitory feedback loop between CDKA;1 and WEE1, seem not to be conserved in *A. thaliana*.

Given all that evidence for G2/M regulation, we integrated the regulatory interactions between stoichiometric CDK inhibitor (KRP1), B-type plant specific CDK and MYB transcriptional factors. It is not surprising that there are clear differences between plant G2 phase regulation and that of other organisms, because variations in this control point could define cell fate. Although differences among the *A. thaliana* CC GRN uncovered here and that of yeasts and animals have now become clear, we think that the basic regulatory CC module reported here, will be a useful framework to incorporate and discover new components of the CC GRNs in plants and also in other eukaryotes.

Despite the fact that our CC GRN model recovers observed CC stage configurations and their canonical pattern of temporal transitions, it did not recover an alternative attractor that corresponds to the endocycle. We hypothesize that the same multi-stable GRN underlies both states, and additional components yet to be connected to the CC GRN will ensure a cyclic attractor corresponding to the complete CC, and another one with shorter period corresponding to the endocycle. In its present form, our model suggests that CYCD3;1 function, which has been associated with the proliferative state [108] and with a delay in the endocycle onset [23], is important to enter the endocycle. Besides, it also has been reported that CYCD3;1 plays a role in G1/S transition [121] and regulates RBR protein during DNA replication [89]. Furthermore, the endoreduplication attractor obtained in some of our mutant simulations (e.g. Fig 5A, 5C and 5D-right) also supports the role of CYCD3;1 in entering an endocycle.

The GRN model of *A. thaliana* CC could help to identify physiological or developmental interactions involved in the tight relationship between proliferation and differentiation observed during different stages of development [1, 88, 108, 109, 126]. Previous to cell division, the cell senses its intracellular and environmental conditions to arrest or promote CC progress. Such cues directly affect the CC machinery, which does not depend on a master or central regulator.

CC control is the result of a network formed by feedback and feedforward loops between complexes of CDK-cyclin and its regulators. It is not evident how complex dynamical processes such as CC progression emerge from simple interactions among components acting simultaneously. The proposed CC GRN will be very helpful to study how cell proliferation/differentiation decisions and balance keeps a suitable spatio-temporal control of CC during plant growth and development.

## Supporting Information

S1 Text Logical rules of *A. thaliana* CC Boolean model. (PDF)

S2 Text Equations, parameters, analysis of parameters and initial conditions of the continuous version of *A. thaliana* CC model. (PDF)

S3 Text New recovered attractors by robustness analysis. Additional attractors yielded by making alterations in each bit of logical functions.
(PDF)

S4 Text Attractors obtained in the simulation of mutant phenotypes. (PDF)
