# Supplementary material for: A Dynamic Gene Regulatory Network Model That Recovers the Cyclic Behavior of Arabidopsis thaliana Cell Cycle
Source: PLoS Comput Biol. 2015 Sep 4;11(9):e1004486. doi: 10.1371/journal.pcbi.1004486 (PMC4560428; doi:10.1371/journal.pcbi.1004486)
Supplement: S1 Text — (PDF) [file pcbi.1004486.s001.pdf]

S1 Text. Logical rules of *A. thaliana* CC Boolean model

$\mathrm{CYCD3;1} = \neg\mathrm{SCF}$
$\mathrm{SCF} = \neg\mathrm{APC/C} \wedge ((\mathrm{E2Fb} \wedge (\neg\mathrm{RBR} \vee (\neg\mathrm{KRP1} \wedge \mathrm{CYCD3;1}))) \vee \mathrm{MYB3R1/4})$
$\mathrm{RBR} = (\mathrm{KRP1} \vee \neg\mathrm{CYCD3;1}) \wedge ((\mathrm{E2Fa} \wedge \neg\mathrm{RBR}) \vee \mathrm{MYB3R1/4})$
$\mathrm{E2Fa} = (\mathrm{E2Fa} \vee \neg\mathrm{E2Fc}) \wedge \neg(\mathrm{CDKB1;1} \wedge \mathrm{CYCA2;3})$
$\mathrm{E2Fb} = (\mathrm{E2Fa} \wedge \neg\mathrm{RBR})$
$\mathrm{E2Fc} = \neg(\mathrm{SCF} \wedge \neg\mathrm{KRP1} \wedge \mathrm{CYCD3;1}) \wedge ((\mathrm{E2Fa} \wedge \neg\mathrm{RBR}) \vee \mathrm{MYB3R1/4})$
$\mathrm{E2Fe} = (\neg\mathrm{E2Fc} \vee (\mathrm{E2Fb} \wedge (\neg\mathrm{RBR} \vee (\neg\mathrm{KRP1} \wedge \mathrm{CYCD3;1})))) \vee \mathrm{MYB77}$
$\mathrm{MYB77} = \mathrm{E2Fb} \wedge (\neg\mathrm{RBR} \vee (\neg\mathrm{KRP1} \wedge \mathrm{CYCD3;1}))$
$\mathrm{MYB3R1/4} = \mathrm{MYB77} \vee (\mathrm{MYB3R1/4} \wedge \mathrm{CYCB1;1} \wedge \neg\mathrm{KRP1})$
$\mathrm{CYCB1;1} = \neg\mathrm{APC/C} \wedge (\mathrm{MYB3R1/4} \vee \mathrm{MYB77} \vee ((\neg\mathrm{RBR} \vee (\neg\mathrm{KRP1} \wedge \mathrm{CYCD3;1})) \wedge \mathrm{E2Fb} \wedge \neg\mathrm{E2Fc}))$
$\mathrm{CDKB1;1} = ((\neg\mathrm{RBR} \vee (\neg\mathrm{KRP1} \wedge \mathrm{CYCD3;1})) \wedge \mathrm{E2Fb} \wedge \neg\mathrm{E2Fc}) \vee \mathrm{MYB3R1/4} \vee \mathrm{MYB77}$
$\mathrm{CYCA2;3} = \neg\mathrm{APC/C} \wedge (\mathrm{MYB3R1/4} \vee \mathrm{MYB77})$
$\mathrm{KRP1} = (\mathrm{MYB77} \vee \mathrm{MYB3R1/4}) \wedge \neg(\mathrm{CDKB1;1} \wedge \mathrm{CYCA2;3} \wedge \mathrm{SCF})$
$\mathrm{APC/C} = \neg\mathrm{E2Fe} \wedge ((\mathrm{E2Fa} \wedge \neg\mathrm{RBR}) \vee \mathrm{MYB3R1/4} \vee \mathrm{MYB77})$
