# Supplementary material for: A Dynamic Gene Regulatory Network Model That Recovers the Cyclic Behavior of Arabidopsis thaliana Cell Cycle
Source: PLoS Comput Biol. 2015 Sep 4;11(9):e1004486. doi: 10.1371/journal.pcbi.1004486 (PMC4560428; doi:10.1371/journal.pcbi.1004486)
Supplement: S2 Text — (PDF) [file pcbi.1004486.s002.pdf]

# S2 Text. Equations, parameters, analysis of parameters and initial conditions of the continuous version of *A. thaliana* CC model

The transformation from logical rules to continuous functions for parameters $\omega_i$ involves the use of fuzzy logic [1, 2], where Boolean operators "AND" ($\wedge$), "OR" ($\vee$), "NOT" ($\neg$) are equivalent to maximum, minimum and $1 - x_i$ fuzzy logic operation, respectively.

## Parameters:

$h = 15$
$\gamma_i = 1$

$\omega_{CYCD3;1} = 1 - SCF$

$\omega_{SCF} = min(1-APC/C, max(min(max(1-RBR, min(1-KRP1, CYCD3;1)), E2Fb), MYB3R1/4))$

$\omega_{RBR} = min(max(KRP1, 1 - CYCD3;1), max(min(E2Fa, 1 - RBR), MYB3R1/4))$

$\omega_{E2Fa} = min(max(E2Fa, 1 - E2Fc), 1 - min(CDKB1;1, CYCA2;3))$

$\omega_{E2Fb} = min(E2Fa, 1 - RBR)$

$\omega_{E2Fc} = min(1 - min(SCF, 1 - KRP1, CYCD3;1), max(min(E2Fa, 1 - RBR), MYB3R1/4))$

$\omega_{E2Fe} = max(1 - E2Fc, min(E2Fb, max(1 - RBR, min(1 - KRP1, CYCD3;1))), MYB77)$

$\omega_{MYB77} = min(E2Fb, max(1 - RBR, min(1 - KRP1, CYCD3;1)))$

$\omega_{MYB3R1/4} = max(MYB77, min(MYB3R1/4, CYCB1;1, 1 - KRP1))$

$\omega_{CYCB1;1} = min(1-APC/C, max(MYB3R1/4, MYB77, min(max(1-RBR, min(1-KRP1, CYCD3;1)), E2Fb, 1-E2Fc)))$

$\omega_{CDKB1;1} = max(min(max(1-RBR, min(1-KRP1, CYCD3;1)), E2Fb, 1-E2Fc), MYB3R1/4, MYB77)$

$\omega_{CYCA2;3} = min(1 - APC/C, max(MYB3R1/4, MYB77))$

$\omega_{KRP1} = min(max(MYB77, MYB3R1/4), 1 - min(CDKB1;1, CYCA2;3, SCF))$

$\omega_{APC/C} = min(1 - E2Fe, max(min(E2Fa, 1 - RBR), MYB3R1/4, MYB77))$

## Rates of change

$$\mathbf{\frac{dx_i}{dt} = \frac{-e^{0.5h} + e^{-h*(\omega_i)}}{(1-e^{0.5h})*(1+e^{-h*(\omega_i - 0.5)})} - \gamma_i x_i}$$

$$\frac{dCYCD3;1}{dt} = \frac{-e^{0.5h} + e^{-h*(\omega_{CYCD3;1})}}{(1-e^{0.5h})*(1+e^{-h*(\omega_{CYCD3;1} - 0.5)})} - CYCD3;1$$

$$\frac{dSCF}{dt} = \frac{-e^{0.5h} + e^{-h*(\omega_{SCF})}}{(1-e^{0.5h})*(1+e^{-h*(\omega_{SCF} - 0.5)})} - SCF$$

$$\frac{dRBR}{dt} = \frac{-e^{0.5h} + e^{-h*(\omega_{RBR})}}{(1-e^{0.5h})*(1+e^{-h*(\omega_{RBR} - 0.5)})} - RBR$$

$$\frac{dE2Fa}{dt} = \frac{-e^{0.5h} + e^{-h*(\omega_{E2Fa})}}{(1-e^{0.5h})*(1+e^{-h*(\omega_{E2Fa} - 0.5)})} - E2Fa$$

$$\frac{dE2Fb}{dt} = \frac{-e^{0.5h} + e^{-h*(\omega_{E2Fb})}}{(1-e^{0.5h})*(1+e^{-h*(\omega_{E2Fb} - 0.5)})} - E2Fb$$

$$\frac{dE2Fc}{dt} = \frac{-e^{0.5h} + e^{-h*(\omega_{E2Fc})}}{(1-e^{0.5h})*(1+e^{-h*(\omega_{E2Fc} - 0.5)})} - E2Fc$$

$$\frac{dE2Fe}{dt} = \frac{-e^{0.5h} + e^{-h*(\omega_{E2Fe})}}{(1-e^{0.5h})*(1+e^{-h*(\omega_{E2Fe} - 0.5)})} - E2Fe$$

$$\frac{dMYB77}{dt} = \frac{-e^{0.5h} + e^{-h*(\omega_{MYB77})}}{(1-e^{0.5h})*(1+e^{-h*(\omega_{MYB77} - 0.5)})} - MYB77$$

$$\frac{dMYB3R1/4}{dt} = \frac{-e^{0.5h} + e^{-h*(\omega_{MYB3R1/4})}}{(1-e^{0.5h})*(1+e^{-h*(\omega_{MYB3R1/4} - 0.5)})} - MYB3R1/4$$

$$\frac{dCYCB1;1}{dt} = \frac{-e^{0.5h}+e^{-h*(\omega_{CYCB1;1})}}{(1-e^{0.5h})*(1+e^{-h*(\omega_{CYCB1;1}-0.5)})} - CYCB1;1$$

$$\frac{dCDKB1;1}{dt} = \frac{-e^{0.5h}+e^{-h*(\omega_{CDKB1;1})}}{(1-e^{0.5h})*(1+e^{-h*(\omega_{CDKB1;1}-0.5)})} - CDKB1;1$$

$$\frac{dCYCA2;3}{dt} = \frac{-e^{0.5h}+e^{-h*(\omega_{CYCA2;3})}}{(1-e^{0.5h})*(1+e^{-h*(\omega_{CYCA2;3}-0.5)})} - CYCA2;3$$

$$\frac{dKRP1}{dt} = \frac{-e^{0.5h}+e^{-h*(\omega_{KRP1})}}{(1-e^{0.5h})*(1+e^{-h*(\omega_{KRP1}-0.5)})} - KRP1$$

$$\frac{dAPC/C}{dt} = \frac{-e^{0.5h}+e^{-h*(\omega_{APC/C})}}{(1-e^{0.5h})*(1+e^{-h*(\omega_{APC/C}-0.5)})} - APC/C$$

## Analysis of parameters:

In this analysis, we assign different values for parameters $h_i$ or $\gamma_i$ of each node $x_i$. While one $h_i$ or one $\gamma_i$ is evaluated, the remaining $hs$ or $\gamma$s takes the default value:

default $h = 15$, default $\gamma = 1$

Set of values for $h_i$: 1, 2, 5, 10, 15, 25, 35, 50, 75 and 100.
Set of values for $\gamma_i$: 1, 1.1, 1.2, 1.3, 1.4, 1.5, 1.6, 1.7, 1.8 and 2.

The same initial condition was used in this analysis: APC/C=1, KRP1=0, CYCA2;3=0, CDKB1;1=1, CYCB1;1=0, MYB3R1/4=1, MYB77=0, E2Fe=0, E2Fc=1, E2Fb=0, E2Fa=0, RBR=1, SCF=0, CYCD3;1=0

Changes in six different $\gamma_i$s cause damped oscillations in the continuous model, and therefore its cyclic behavior is lost. These were: $\gamma_{APC/C}$, $\gamma_{CYCD3;1}$, $\gamma_{E2Fa}$, $\gamma_{E2Fb}$, $\gamma_{E2Fc}$ and $\gamma_{MYB77}$. The cyclic attractor also was lost in four $h_i$s: $h_{E2Fc}$, $h_{KRP1}$, $h_{MYB3R1/4}$ and $h_{SCF}$. In total, only 7.14% of the cases (i.e. 20 of the 280 cases; 28 parameters with 10 possible values) did not maintain the cyclic attractor, while the remaining 92.86% maintained the same cyclic attractor. The 20 cases that modified the attractor are shown below:

**Parameter 1.** $\gamma_{APC/C} = 2$

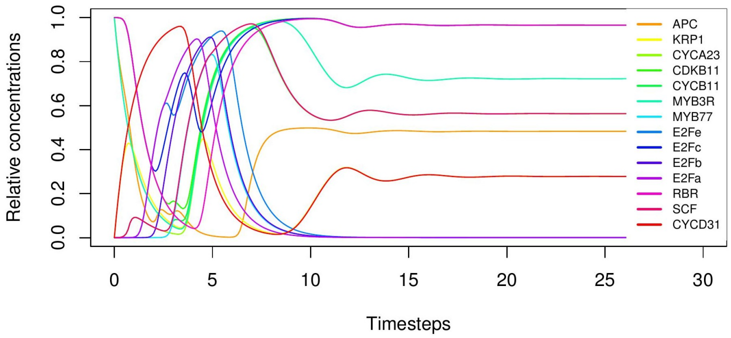

**Parameter 2.** $\gamma_{CYCD3;1} = 2$

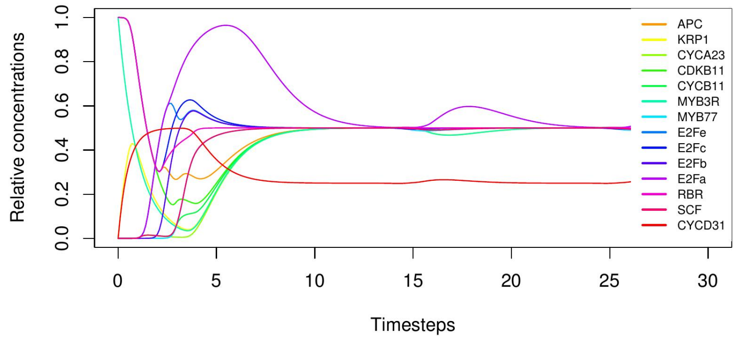


**Parameter 3.** $\gamma_{E2Fa} = 1.4, 1.5, 1.6, 1.7, 1.8, 2$

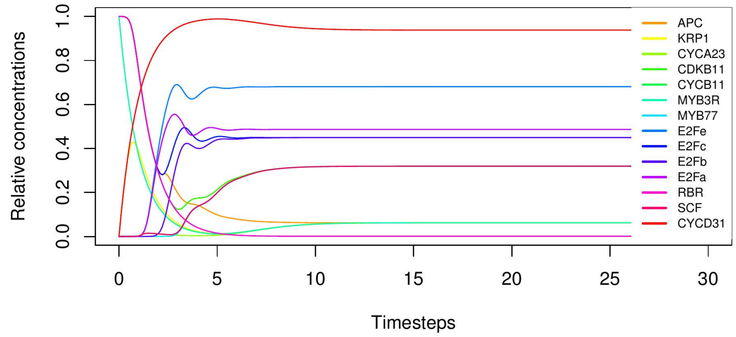


**Parameter 4.** $\gamma_{E2Fb} = 2$

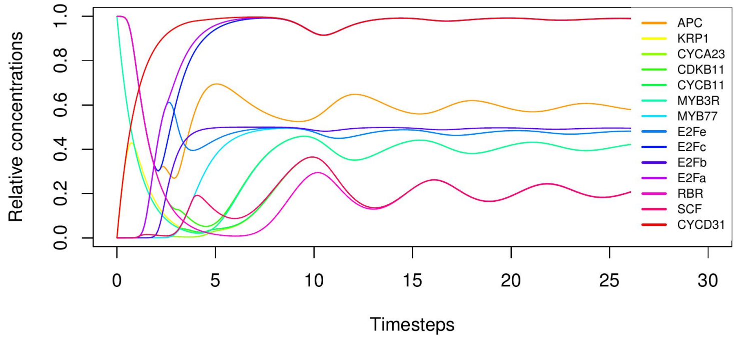


**Parameter 5.** $\gamma_{E2Fc} = 2$

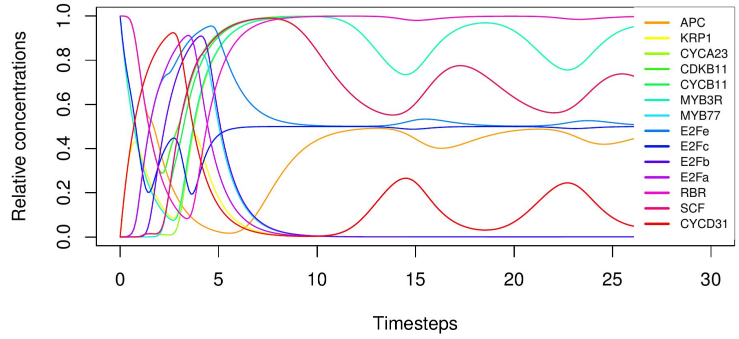

**Parameter 6.** $\gamma_{MYB77} = 1.7, 1.8, 2$

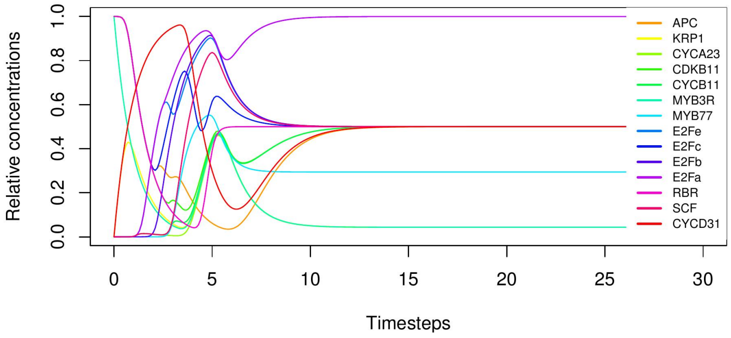


**Parameter 7.** $h_{E2Fc} = 1, 2$

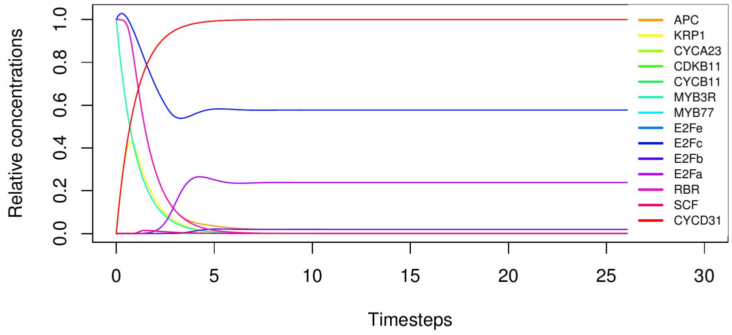


**Parameter 8.** $h_{KRP1} = 1, 2$

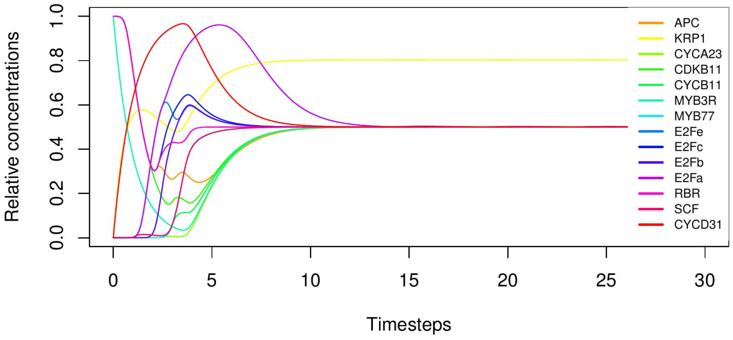


**Parameter 9.** $h_{MYB3R1/4} = 1$

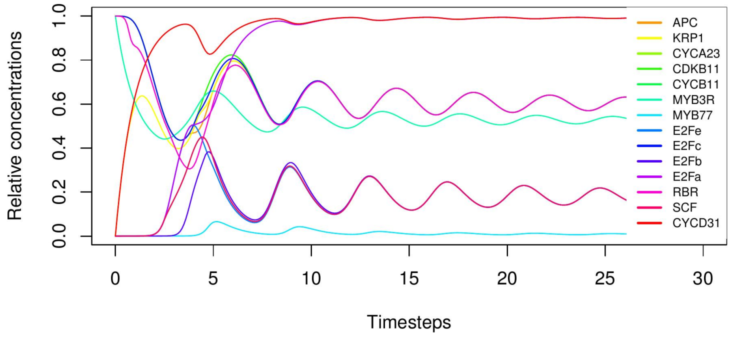

**Parameter 10.** $h_{SCF} = 1, 2$

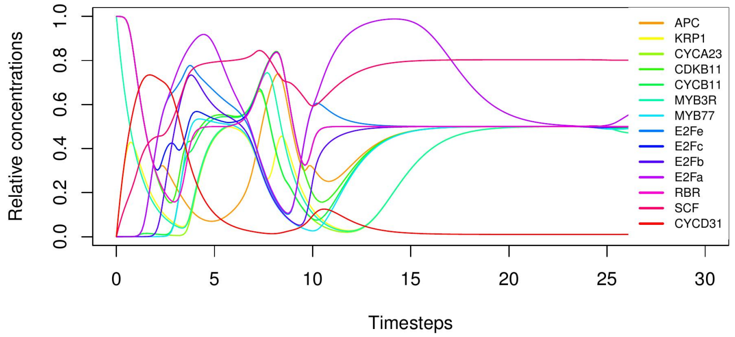


## 1000 initial conditions used to find the steady states of the continuous model

ORDER: APC/C KRP1 CYCA2;3 CDKB1;1 CYCB1;1 MYB3R1/4 MYB77 E2Fe E2Fc E2Fb E2Fa RBR SCF CYCD3;1

| | | | | |
|---|---|---|---|---|
| 00100000010010 | 01110011101001 | 00001100101001 | 11101010001110 | 00111010101001 |
| 10000100110111 | 10000101100101 | 00110001110110 | 11011011011010 | 00010011011110 |
| 10100100001110 | 01000111001000 | 01000011011101 | 00000000101100 | 10110001011001 |
| 11110011111000 | 10100101000111 | 01011010110101 | 01001111000100 | 10000010110100 |
| 10111100011101 | 10000111100110 | 00100001010110 | 00110101110001 | 10101100111010 |
| 10010000100110 | 01111010111010 | 00001111010100 | 00100110101110 | 01010001110000 |
| 10111010100110 | 11010010011000 | 11010011111010 | 00101111011011 | 10111110111011 |
| 00011100010011 | 01010000010000 | 00101001101000 | 11101000111000 | 00011010010001 |
| 00101010111111 | 10111011111111 | 10010110111011 | 10011010110101 | 01110011100110 |
| 01111001101001 | 01000010000101 | 10000111110011 | 00101011110101 | 01111110001001 |
| 10100011001010 | 00111011011101 | 01001000100101 | 11001011011111 | 00100010101100 |
| 00101000100110 | 00011010100111 | 10111100111011 | 01101010100000 | 01110100000011 |
| 11000001111000 | 01110010000100 | 00110011011000 | 00100100010110 | 10010001001001 |
| 00011100110100 | 01100011100111 | 01100010110011 | 11101111111001 | 11011110101011 |
| 10001010111111 | 01100001010001 | 01110010100101 | 00100001101110 | 01111010101000 |
| 11001000111001 | 00011010101110 | 11100001111110 | 10101011001001 | 10111110011010 |
| 01110000001000 | 10111001100101 | 10001101100000 | 10001001010011 | 11111111111100 |
| 01001011110101 | 00101011101110 | 00000000010101 | 10110110111001 | 10100001111001 |
| 10101011111000 | 01001000111011 | 00000101110001 | 00001010001001 | 10010001000010 |
| 11011110000010 | 01100111111110 | 10000110111010 | 01100001010010 | 11000110101001 |
| 10101101111011 | 11100101110101 | 11111111010110 | 01010000101110 | 11100110001111 |
| 00100011101110 | 10000001010001 | 01101000001100 | 10000000001100 | 10110110010000 |
| 11000001100001 | 10011010110000 | 11100001111011 | 11010000110111 | 00110001011011 |
| 01100000100111 | 00110001110011 | 10110011010010 | 00011110110001 | 11111010000001 |
| 01001111111001 | 01101101010010 | 10100000101111 | 01011101001111 | 01010111100001 |
| 11100001100000 | 11011011000000 | 10100011100101 | 11110001011011 | 11011001000010 |
| 11111100111000 | 11011101101100 | 10110011011011 | 00000001010010 | 11001101011101 |
| 10001100110010 | 00100010101110 | 11101011010111 | 01010111110011 | 11110011001111 |
| 01011000111101 | 00001011001111 | 10110100011001 | 00100000001100 | 10111101011000 |
| 00001110111110 | 11110100010001 | 01110110110011 | 10000010110111 | 11110010010101 |
| 11000111100111 | 11010111010011 | 00100111101000 | 01100000101111 | 10010000001110 |
| 11001111011111 | 01100000010000 | 01011011001101 | 01011111101000 | 01100011101110 |
| 00111000111100 | 10010000110100 | 00000011110001 | 01011110100000 | 11101010011111 |
| 11011100110010 | 00011011011110 | 00010001000001 | 01111101100100 | 00101011100011 |
| 01000111000011 | 10010000011000 | 01001100110100 | 01100010011100 | 11100110000100 |
| 01001101000101 | 00010010010100 | 11111100010101 | 11010010000110 | 11000011010011 |

| | | | | |
|---|---|---|---|---|
| 11100000110111 | 10101001101101 | 10000100001110 | 01110000000001 | 00111111010010 |
| 11000100111010 | 11101110010001 | 11010000110000 | 11101110110110 | 11100111101110 |
| 11011000011111 | 10100111000110 | 01100011111000 | 01001000110101 | 10100010010111 |
| 01100100010100 | 10010011110111 | 01101100010100 | 01000101101011 | 10101001111101 |
| 01101100100110 | 10110000101010 | 10110101001010 | 10011110101100 | 01100110000011 |
| 11100010011111 | 10100001110110 | 01010011000101 | 11101100000001 | 00110101110111 |
| 00101111100000 | 10111011010000 | 10111001111100 | 01001110101001 | 10110001010110 |
| 10110001010111 | 01011011011000 | 10010101101001 | 00101000001001 | 10101001101010 |
| 01010011000111 | 11111110111000 | 01110000001111 | 00001101101100 | 11000011100000 |
| 01100110000111 | 01101111111010 | 11110110000100 | 11110101011100 | 01011001011101 |
| 10101011110101 | 10101001101011 | 00111001100011 | 10110000011000 | 00011110101100 |
| 10101101000101 | 10001100010111 | 01100010101101 | 10000011011110 | 01001001111000 |
| 11100110001010 | 00000010111011 | 01011001111011 | 10000101000001 | 01111111101110 |
| 10000111101000 | 01001110000001 | 11111001101000 | 01110110101100 | 00111110110010 |
| 11100101111101 | 00110110111010 | 01110001001011 | 00010010011110 | 00001100111101 |
| 01110101111100 | 01011010011100 | 00110011010111 | 00010111000101 | 01011111001101 |
| 01110000010101 | 10001000101011 | 11000001001100 | 01110001000101 | 10100000100100 |
| 10111110111110 | 00000001010111 | 00111100111010 | 00011010001011 | 10010001010111 |
| 01010010000010 | 11100100000011 | 01010110101100 | 11100010001001 | 00110100110101 |
| 01010100101011 | 10100000011001 | 10100010011000 | 00001001001001 | 11111100100000 |
| 11101101000001 | 10101011110010 | 11110100101101 | 10011110101010 | 11100011011001 |
| 11110000010100 | 00000000100011 | 10001001101101 | 01111000010001 | 11101111000001 |
| 00001110000100 | 11111111001110 | 00100011101001 | 11010111001001 | 11110000110001 |
| 10111010001110 | 00101001000101 | 10010111010001 | 00110000101111 | 11010001001101 |
| 11001111000110 | 00110111101111 | 11011111110101 | 11011100101010 | 00100011011000 |
| 01001001010010 | 01010001100001 | 11101101011000 | 00101010000101 | 11010001101100 |
| 00101000001011 | 11111000110101 | 10111001110001 | 00000110011011 | 01000011001001 |
| 01010000010101 | 01001110000000 | 11000001010011 | 10100001111010 | 11101010011101 |
| 00001011011011 | 01000101000001 | 00111100001110 | 01101110010101 | 11000010000111 |
| 10110110101101 | 11100011000101 | 11010011011110 | 00100010100110 | 11100101100110 |
| 10001010100000 | 11100110101001 | 01000100110111 | 01001011100101 | 01111100011110 |
| 00110001111110 | 01110110000110 | 11010111110110 | 00010111001000 | 00001001110110 |
| 11110101000010 | 10111110100010 | 11011000000100 | 11111110101111 | 00101100011110 |
| 11100101101000 | 01001001110100 | 00110011001000 | 10101000001111 | 10101101001010 |
| 01011111001111 | 01101010101100 | 01100001001100 | 00010110011011 | 00101110001001 |
| 10110100101010 | 01100111011101 | 00111111111000 | 01110100101101 | 11101111110011 |
| 00001001001100 | 00010110000100 | 11111000110000 | 11100110000000 | 01101001100000 |
| 10000010011000 | 11110001101100 | 10010001100100 | 11100000101001 | 01001011100110 |
| 01100101100010 | 01010110111010 | 11011101000110 | 10010100000000 | 01000101010001 |
| 11111111000111 | 10011101001101 | 01101011001010 | 10100001010111 | 01100111100111 |
| 11010100010000 | 01001100010010 | 00000111100001 | 10000010000010 | 01101000010110 |
| 01000000001010 | 00101110101110 | 01001001111110 | 11101010110111 | 10000000010010 |
| 00111101110100 | 10001010110101 | 00000101000111 | 10111110001010 | 00100011001111 |
| 00100101101101 | 00000100101011 | 00011111101000 | 00010000100101 | 01010000000011 |
| 00110111010001 | 11100011101000 | 11001110000101 | 01001110011000 | 10010100101001 |
| 00001100011101 | 01111111000111 | 10000110100110 | 01101100111001 | 10101000011010 |
| 01011010110001 | 01001101111010 | 01010010011101 | 01100010111111 | 00001100001101 |
| 00110011011100 | 10001100000010 | 11110010101011 | 01000011000111 | 11010010100010 |
| 00100000100110 | 10010101010001 | 10110001110011 | 10000001010010 | 10001111010010 |
| 01110010000101 | 10001001011000 | 10011000101101 | 01010111010110 | 10000001010110 |
| 01110100011111 | 10010000110110 | 00000101101011 | 00010100000000 | 10110010101000 |
| 11101110111111 | 00110001100000 | 01110100110001 | 00100110011101 | 01110001110000 |
| 00000010100011 | 01100101110101 | 11001010111101 | 01100101101000 | 00111010011101 |
| 01101111000000 | 01011110010011 | 00011011001001 | 10100000011010 | 00101011011011 |

| | | | | |
|---|---|---|---|---|
| 01111011001100 | 11110011110101 | 00100001001100 | 01111010100011 | 00000111000100 |
| 01111011101001 | 00000011111100 | 00011111001100 | 11100001011001 | 01000100000111 |
| 10011110100101 | 01001101110111 | 11101100111111 | 10100011011001 | 00000110010001 |
| 00100011100000 | 11110110111111 | 11000011100100 | 10010000010101 | 01010111000000 |
| 01110100110111 | 01100111111011 | 01001011101101 | 10000011100000 | 01010101011101 |
| 10111111101101 | 10001101011011 | 10011100100110 | 10001100100011 | 00010000001111 |
| 10000010111011 | 01000111110111 | 01100000001011 | 11010000111011 | 11001110000001 |
| 01100001101100 | 00111110110101 | 11010101011010 | 01101111100110 | 01001001000000 |
| 01101101001011 | 01111011101010 | 10111011110110 | 01110110010110 | 01010001010000 |
| 10001011011010 | 00110110110100 | 10101011111010 | 10011110000110 | 00111001111101 |
| 01100101111011 | 11111110100100 | 10000001101000 | 00111011101110 | 01100010110010 |
| 10011010000110 | 11001110110000 | 01101100001010 | 01010010000011 | 00011101000010 |
| 00100011001000 | 11011011111101 | 00111111100011 | 00110011101100 | 11100010001110 |
| 01010000110000 | 10110001111100 | 11001100001010 | 11101001011001 | 10110000110000 |
| 01111000101111 | 00101001111111 | 11001110000110 | 01011101101000 | 10110001100001 |
| 11000010010111 | 01100001100001 | 01000010100101 | 00110101010011 | 11000100101010 |
| 10000100001001 | 10111110111000 | 01001100011010 | 10101111101111 | 01000001100010 |
| 01011010100000 | 11101110101100 | 00010101010011 | 11111101100110 | 11111101100111 |
| 11101111011000 | 11000110100110 | 11101100010111 | 10110101110110 | 01001001101100 |
| 11000101011001 | 01100110111111 | 01100100000011 | 00010001101100 | 01011001000111 |
| 11101111100010 | 00010100101001 | 00100100000001 | 11111011011000 | 11010111000110 |
| 01101010011111 | 00001100111110 | 11110011111101 | 10111000000111 | 00110000111101 |
| 01000001011111 | 00111110101101 | 10110000111111 | 11010100001101 | 00001011111011 |
| 11010001000000 | 11001110100110 | 11111110110000 | 10101100101001 | 11110101101111 |
| 10110011110101 | 11111111000100 | 11100101110010 | 00001001111000 | 00010001011010 |
| 01101001000001 | 10010101110001 | 00100111100100 | 10001011000110 | 11000010110101 |
| 01010101000001 | 11000000001101 | 10110111010001 | 10010010011001 | 11111010101100 |
| 00100010011100 | 10010010011111 | 01110100101011 | 00101001010101 | 10010111100010 |
| 11111101111010 | 10000000111011 | 00111000101001 | 11101110110001 | 10111001100010 |
| 00000111100101 | 10100010101110 | 11011000011011 | 00101110011011 | 00011111011010 |
| 10101110010010 | 10001000011111 | 00000011110110 | 11010111000101 | 01001000011010 |
| 10001111111100 | 11011100000011 | 10010111011001 | 11001100111111 | 00001010110010 |
| 10101111110100 | 11110100001111 | 10101100100101 | 11010100011011 | 11110000001010 |
| 01111100010111 | 11001010110110 | 11000100000101 | 10000110100111 | 11111110010111 |
| 00001101110111 | 00001010101001 | 10001010011101 | 10100100111101 | 10010001011110 |
| 10101001010010 | 10111111100110 | 10101010101111 | 10010110001101 | 00101000101101 |
| 11101110000101 | 10001101101101 | 00000110100010 | 10110000101000 | 00011010110110 |
| 00111100000010 | 00110011101001 | 11101111000100 | 11101100000011 | 00101011101010 |
| 00011101000011 | 01001011010010 | 10000011110000 | 10010101010000 | 01101000110110 |
| 11111001001001 | 01101100111100 | 11111011101000 | 10011111011001 | 01000111101110 |
| 11010100110011 | 00101111111011 | 11101111100110 | 00001110010000 | 10110010011010 |
| 00101100001101 | 11111100011110 | 01100011011111 | 01101111010000 | 10010001011111 |
| 11100000011010 | 11000000111110 | 10111101010010 | 10011100101000 | 01000101000101 |
| 11110111001111 | 10110010001101 | 01001000010100 | 00111111011101 | 01001011111110 |
| 11010111000000 | 00111101111000 | 11010101011110 | 11110110101110 | 01101001000110 |
| 01111100110010 | 10101000110010 | 10010100001111 | 11010011101000 | 10111001001111 |
| 10101101101001 | 11000010110110 | 10100010000011 | 10010011001000 | 11010111111101 |
| 11111000100101 | 11101001001011 | 01010000110110 | 10100010100100 | 00110010111111 |
| 00100001010111 | 00010111111111 | 00111101111100 | 10010110001001 | 00101010111110 |
| 10100111010111 | 01110010011101 | 01010010010011 | 00111101001010 | 11011100100110 |
| 01111001001010 | 10111110001001 | 11101000000011 | 11101100010101 | 01001100000101 |
| 00011100101010 | 01010001011011 | 01000000000011 | 10001011110001 | 11011000111110 |
| 01000010011010 | 00111000001001 | 01010111111010 | 00000001001011 | 01010000101001 |
| 00101101000001 | 01011111101110 | 10100111001101 | 10110000100101 | 11110000011111 |

| | | | | |
|---|---|---|---|---|
| 11000101011000 | 11100101111011 | 10110111111011 | 11111110101000 | 11110100111110 |
| 01000000011100 | 01001010011000 | 10110100110100 | 00010010000100 | 11111010111010 |
| 01010000100111 | 10011010000001 | 01110100111011 | 01101011100100 | 01101110001010 |
| 11001111011100 | 00110010110101 | 00001000110010 | 00011101001010 | 10000101111100 |
| 01101110111111 | 00111000110011 | 11111111011100 | 10001100100001 | 11001010101001 |
| 00111101111111 | 00000110100110 | 11001011111011 | 01100011001100 | 11000000101011 |
| 00110010001001 | 00110001000101 | 00011100100110 | 10111101011110 | 10100001111011 |
| 11110011111001 | 01010110001001 | 10101000101011 | 01011001100110 | 01011010111101 |
| 00111101001100 | 01101110111101 | 01110000001110 | 01000011001111 | 10111001111101 |
| 10001100101011 | 10101101010111 | 10101001111111 | 01111110110000 | 00000110100000 |
| 10010101101000 | 11111011111100 | 11010011001000 | 00001011011101 | 10000111110010 |
| 01010001011000 | 10101111101100 | 11001000100111 | 10011111001010 | 00000010010110 |
| 00010010000001 | 10011100000110 | 01000010001101 | 00100100010100 | 11100011101100 |
| 01101110110110 | 11000110111111 | 10101110101011 | 10011000110111 | 00011000101100 |
| 10110010111110 | 10001100011100 | 01111111110001 | 01110010110100 | 00101101111000 |
| 11010010011010 | 01110101101001 | 00100011111001 | 10100100011000 | 10101100000100 |
| 11000001111100 | 01101010111101 | 10001111110101 | 11011100000100 | 01000000101000 |
| 00011110101000 | 11111001101111 | 11110100101111 | 00101010100110 | 01110000101100 |
| 01011100111010 | 01011100010111 | 11100110100100 | 01110010110001 | 00001111010111 |
| 11100001010010 | 11100110110011 | 00001111100011 | 10001101011110 | 10100011010011 |
| 11101010111111 | 11110011001001 | 00110000101010 | 00010111100110 | 11100000100011 |
| 00101001100011 | 11010110110000 | 10001001000111 | 11011011000010 | 11100010101001 |
| 11100001111010 | 11010101010110 | 11001111101110 | 10010100000001 | 11100001011110 |
| 11110111001110 | 11100010110100 | 11010111111011 | 01100111111001 | 10111110001110 |
| 11011101101101 | 01111011111101 | 10001100010010 | 11101110100101 | 11110010101110 |
| 01100010100110 | 01101001100101 | 00000000011011 | 00011111110011 | 00110100101011 |
| 00100100111010 | 11111111110011 | 11111100011111 | 00010011101000 | 11011111100110 |
| 10111111101111 | 00111000001110 | 00001001100111 | 01111100000000 | 11001011001101 |
| 01100001101001 | 00010001100110 | 00100011111111 | 10101100110110 | 01000010011001 |
| 11101010110101 | 01100110010101 | 00011110011110 | 11011011101100 | 11011001101011 |
| 00000001001100 | 10010100101111 | 01010100110001 | 01000110111001 | 10111011100100 |
| 01000011010110 | 10011000010001 | 10100111001001 | 01100101001001 | 11001101100100 |
| 00101000100111 | 00010000000011 | 00000000111101 | 01110101011101 | 00111011100101 |
| 00011110101001 | 10010100001011 | 10111110011000 | 11001111101100 | 00100101100010 |
| 11101100001111 | 11000010101101 | 11111010001110 | 01100011011010 | 10111000101101 |
| 10100111011111 | 11110011001100 | 11101101011111 | 01111101000110 | 10111111001000 |
| 11011111001111 | 01010100100001 | 10110100111010 | 10001100000101 | 11010010110110 |
| 01011100010001 | 01001101010010 | 01100000101110 | 11110101010011 | 01100111110000 |
| 10111010010001 | 11100000110100 | 01011011000010 | 11001100111001 | 00001011101100 |
| 11100110111101 | 10110001101110 | 01111111000001 | 00111011001101 | 00001101001100 |
| 00011111010110 | 01100011001010 | 11111001000100 | 11011001010010 | 11100010011010 |
| 01011101110101 | 01000010000110 | 00011111111111 | 00001100011010 | 01010111011111 |
| 11010110011000 | 00101000110111 | 00011110001110 | 11011000010111 | 00000000010010 |
| 01000101001011 | 10110010101111 | 11100000010100 | 00100000001110 | 11101000010001 |
| 11111000000001 | 00011101101111 | 10101111001100 | 01011011101111 | 01011000100101 |
| 01000110011101 | 01010101001110 | 01011100101101 | 00100100110101 | 00110000111100 |
| 10110111001011 | 11110000010011 | 00110011110101 | 11100010111110 | 00011101101100 |
| 10001101011000 | 11110110001000 | 11110001010110 | 11000001000101 | 11010001011100 |
| 10110111111110 | 01111111001011 | 01001111101101 | 11010001000010 | 01100110001001 |
| 11011111101101 | 01100000101001 | 11011010010101 | 11000101000100 | 01110110010111 |
| 10011001100111 | 01100010011001 | 10111001011010 | 01111110101010 | 00110000010011 |
| 01101101111110 | 01001100101110 | 11111001111110 | 11110000011000 | 01111111111000 |
| 10100001101101 | 00011000001100 | 11100011110000 | 01010000000101 | 00111010110010 |
| 01010110111011 | 01011100000110 | 11001000111101 | 11001010101111 | 10010101101100 |

| | | | |
|---|---|---|---|
| 01001001101011 | 00100011010000 | 10100010100001 | 00111100000100 |
| 00100111010101 | 11001110011000 | 10000011011010 | |
| 10100100110110 | 01000100110011 | 11110010000010 | |

**For the transformation from Boolean to continuous model, we also employ Odefy, a method that uses hill functions ([118,119] from the main reference list). The sustained oscillatory behavior of CC molecular components was also recovered:**

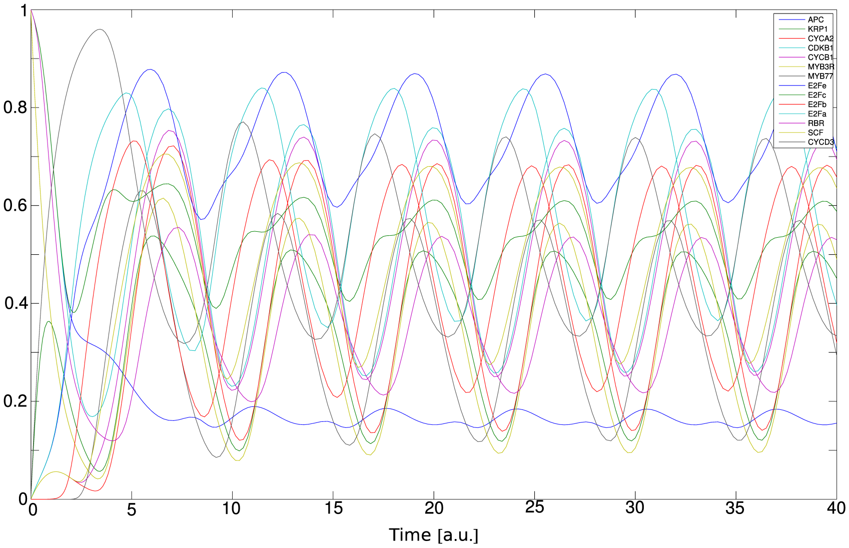


# References


1. Weinstein N, Mendoza L. Building Qualitative Models of Plant Regulatory Networks with SQUAD. Front Plant Sci. 2012;3:72.

2. Villarreal C, Padilla-Longoria P, Alvarez-Buylla ER. General theory of genotype to phenotype mapping: derivation of epigenetic landscapes from N-node complex gene regulatory networks. Phys Rev Lett. 2012 9;109(11):118102.
