# Supplementary material for: A Dynamic Gene Regulatory Network Model That Recovers the Cyclic Behavior of Arabidopsis thaliana Cell Cycle
Source: PLoS Comput Biol. 2015 Sep 4;11(9):e1004486. doi: 10.1371/journal.pcbi.1004486 (PMC4560428; doi:10.1371/journal.pcbi.1004486)
Supplement: S3 Text — Additional attractors yielded by making alterations in each bit of logical functions. (PDF) [file pcbi.1004486.s003.pdf]

S3 Text. New recovered attractors by robustness analysis.

From the output of the truth tables, we change one bit at a time, and then we see the effect that they have on the original attractor. In this supplementary file, we present the thirteen cases where the original attractor was modified by increasing period (six cases) or where additional attractors were found (seven cases). The size of the basin of attraction of the new or the modified attractors is indicated, as well as the percentage that such size represents on the total number of network configurations.

For the attractors, the order of nodes is: APC/C, KRP1, CYCA2;3, CDKB1;1, CYCB1;1, MYB3R1/4, MYB77, E2Fe, E2Fc, E2Fb, E2Fa, RBR, SCF and CYCD3;1.

1. Bit changed in the truth table for SCF

APC/C	KRP1	MYB3R1/4	E2Fb	RBR	CYCD3;1	SCF
0	0	0	0	1	0	1

Cyclic attractor period 9
Basin of attraction of 3722 network configurations (22.72%):

00000001001000
00000001111101
00000011001011
01111101011000
01111011110111
00111101000000
01111101100111
00111000100110
00000000000010

2. Bit changed in the truth table for RBR

KRP1	MYB3R1/4	E2Fa	RBR	CYCD3;1	RBR
0	0	1	1	1	1

Fixed-point attractor
Basin of attraction of 48 network configurations (0.29%): 00000001001101

3. Bit changed in the truth table for RBR

KRP1	MYB3R1/4	E2Fa	RBR	CYCD3;1	RBR
0	0	0	0	1	1

Cyclic attractor period 12
Basin of attraction of 16384 network configurations (100%):

00000001001001
00000001111001
00000011111011
01111111011010
00111111110110
00111101100110
00111100100110
10111100100110
10010100100100
11010000100101
00000000000001
00000001001101

4. Bit changed in the truth table for E2Fa

CYCA2;3	CDKB1;1	E2Fc	E2Fa	E2Fa
1	1	1	1	1

Cyclic attractor period 10
Basin of attraction of 3257 network configurations (19.88%):

10000001111001
00000011111001
01111111111011
00111111111110
00111101101110
00111100101110
10111100101110
10010100101100
11010000101101
00000000001001

5. Bit changed in the truth table for E2Fc

KRP1	MYB3R1/4	E2Fa	RBR	SCF	CYCD3;1	E2Fc
1	0	0	1	0	1	1

Cyclic attractor period 12
Basin of attraction of 16384 network configurations (100%):

00000001001001
00000001111001
00000011111011
01111111011010
00111111110110
00111101100110
00111100100110
10111100100110
10010100100100
11010000100101
00000000100001
00000000000001

6. Bit changed in the truth table for MYB77

KRP1	E2Fb	RBR	CYCD3;1	MYB77
1	1	1	1	1

Cyclic attractor period 3
Basin of attraction of 2017 network configurations (12.31%):

11111101001001
01010001110101
00000010000001

7. Bit changed in the truth table for MYB77

KRP1	E2Fb	RBR	CYCD3;1	MYB77
1	0	1	1	1

Cyclic attractor period 14
Basin of attraction of 12760 network configurations (77.88%):

00000001001001
00000001111001
00000011111011
01111111011010
00111111110110
00111101100110
00111100100110
10111100100110
10010100100100
11010000100101
00000010000001
11111101001001
01010001110101
00000000000001

Cyclic attractor period 3
Basin of attraction of 3624 network configurations (22.12%):

01010101100101
01111010100111
10111111000000

8. Bit changed in the truth table for MYB77

KRP1	E2Fb	RBR	CYCD3;1	MYB77
0	1	1	0	1

Cyclic attractor period 12
Basin of attraction of 16384 network configurations (100%):

00000001001001
00000001111001
00000011111011
01111111011010
00111111110110
00111111100110
00111101100110
00111100100110
10111100100110
10010100100100
11010000100101
00000000000001

9. Bit changed in the truth table for MYB3R1/4

KRP1	CYCB1;1	MYB3R1/4	MYB77	MYB3R1/4
1	1	0	0	1

Cyclic attractor period 2
Basin of attraction of 6547 network configurations (39.96%):

00000100000000
11111001101111

10. Bit changed in the truth table for MYB3R1/4

KRP1	CYCB1;1	MYB3R1/4	MYB77	MYB3R1/4
1	0	1	0	1

Fixed-point attractor 1
Basin of attraction of 81 network configurations (0.49%): 11010100100101

Fixed-point attractor 2
Basin of attraction of 57 network configurations (0.35%): 11010100101101

11. Bit changed in the truth table for MYB3R1/4

KRP1	CYCB1;1	MYB3R1/4	MYB77	MYB3R1/4
1	0	0	0	1

Cyclic attractor period 14
Basin of attraction of 16384 network configurations (100%):

00000001001001
00000001111001
00000011111011
01111111011010
00111111110110
00111101100110
00111100100110
10111100100110
10010100100100
11010000100101
00000100000001
11111001101011
00000000110100
00000000000001

12. Bit changed in the truth table for MYB3R1/4

KRP1	CYCB1;1	MYB3R1/4	MYB77	MYB3R1/4
0	0	1	0	1

Cyclic attractor period 12
Basin of attraction of 16384 network configurations (100%):

00000001001001
00000001111001
00000011111011
01111111011010
00111111110110
00111101100110
00111100100110
10111100100110
10010100100100
11010100100101
11010000100101
00000000000001

13. Bit changed in the truth table for KRP1

CYCA2;3	CDKB1;1	MYB3R1/4	MYB77	SCF	KRP1
1	1	1	0	0	0

Cyclic attractor period 3
Basin of attraction of 1385 network configurations (8.45%):

01111101001000
00111001110111
00000011000010
