# Supplementary material for: A Dynamic Gene Regulatory Network Model That Recovers the Cyclic Behavior of Arabidopsis thaliana Cell Cycle
Source: PLoS Comput Biol. 2015 Sep 4;11(9):e1004486. doi: 10.1371/journal.pcbi.1004486 (PMC4560428; doi:10.1371/journal.pcbi.1004486)
Supplement: S4 Text — (PDF) [file pcbi.1004486.s004.pdf]

S4 Text. Attractors obtained in the simulation of mutant phenotypes

Wild-Type

Attractor(s) with period 11

	Attr. 1										
**APC/C**	1	1	0	0	0	0	0	0	0	0	1
**KRP1**	0	1	0	0	0	0	1	0	0	0	0
**CYCA2;3**	0	0	0	0	0	0	1	1	1	1	1
**CDKB1;1**	1	1	0	0	0	0	1	1	1	1	1
**CYCB1;1**	0	0	0	0	0	0	1	1	1	1	1
**MYB3R1/4**	1	0	0	0	0	0	1	1	1	1	1
**MYB77**	0	0	0	0	0	1	1	1	0	0	0
**E2Fe**	0	0	0	1	1	1	1	1	1	0	0
**E2Fc**	1	1	0	0	1	1	0	1	1	1	1
**E2Fb**	0	0	0	0	1	1	1	1	0	0	0
**E2Fa**	0	0	0	1	1	1	1	0	0	0	0
**RBR**	1	1	0	0	0	0	0	1	1	1	1
**SCF**	0	0	0	0	0	1	1	1	1	1	1
**CYCD3;1**	0	1	1	1	1	1	0	0	0	0	0
Freq.	100%										

APC/C loss of function

	Fixed-point attractor(s)
	Attr. 1
**APC/C**	0
**KRP1**	0
**CYCA2;3**	1
**CDKB1;1**	1
**CYCB1;1**	1
**MYB3R1/4**	1
**MYB77**	0
**E2Fe**	0
**E2Fc**	1
**E2Fb**	0
**E2Fa**	0
**RBR**	1
**SCF**	1
**CYCD3;1**	0
Freq.	100%

APC/C gain of function

Attractor(s) with period 7							
	Attr. 1						
**APC/C**	1	1	1	1	1	1	1
**KRP1**	0	0	0	1	1	1	1
**CYCA2;3**	0	0	0	0	0	0	0
**CDKB1;1**	0	0	0	1	1	1	1
**CYCB1;1**	0	0	0	0	0	0	0
**MYB3R1/4**	0	0	0	1	1	1	0
**MYB77**	0	0	1	1	1	0	0
**E2Fe**	0	1	1	1	1	1	0
**E2Fc**	0	1	1	1	1	1	1
**E2Fb**	0	1	1	1	1	0	0
**E2Fa**	1	1	1	1	1	1	1
**RBR**	0	0	0	0	1	1	1
**SCF**	0	0	0	0	0	0	0
**CYCD3;1**	1	1	1	1	1	1	1
Freq.	100%						

KRP1 loss of function

Attractor(s) with period 11											
	Attr. 1										
**APC/C**	1	1	0	0	0	0	0	0	0	0	1
**KRP1**	0	0	0	0	0	0	0	0	0	0	0
**CYCA2;3**	0	0	0	0	0	0	1	1	1	1	1
**CDKB1;1**	1	1	0	0	0	0	1	1	1	1	1
**CYCB1;1**	0	0	0	0	0	0	1	1	1	1	1
**MYB3R1/4**	1	0	0	0	0	0	1	1	1	1	1
**MYB77**	0	0	0	0	0	1	1	1	0	0	0
**E2Fe**	0	0	0	1	1	1	1	1	1	0	0
**E2Fc**	1	1	0	0	1	1	0	1	1	1	1
**E2Fb**	0	0	0	0	1	1	1	1	0	0	0
**E2Fa**	0	0	0	1	1	1	1	0	0	0	0
**RBR**	1	1	0	0	0	0	0	1	1	1	1
**SCF**	0	0	0	0	0	1	1	1	1	1	1
**CYCD3;1**	0	1	1	1	1	1	0	0	0	0	0
Freq.	100%										

KRP1 gain of function

	Attractor(s) with period 2	
	Attr. 1	
**APC/C**	0	1
**KRP1**	1	1
**CYCA2;3**	0	0
**CDKB1;1**	0	0
**CYCB1;1**	0	0
**MYB3R1/4**	0	0
**MYB77**	0	0
**E2Fe**	0	1
**E2Fc**	0	1
**E2Fb**	0	1
**E2Fa**	1	1
**RBR**	0	1
**SCF**	0	0
**CYCD3;1**	1	1
Freq.	100%	

CYCA2;3 loss of function

Attractor(s) with period 7

	Attr. 1						
**APC/C**	0	1	0	0	0	0	0
**KRP1**	0	0	0	1	1	1	1
**CYCA2;3**	0	0	0	0	0	0	0
**CDKB1;1**	0	0	0	1	1	1	1
**CYCB1;1**	0	0	0	1	1	1	1
**MYB3R1/4**	0	0	0	1	0	1	0
**MYB77**	0	0	1	0	1	0	0
**E2Fe**	0	1	1	1	1	1	1
**E2Fc**	0	1	0	1	1	0	1
**E2Fb**	0	1	0	1	1	0	1
**E2Fa**	1	1	1	1	1	1	1
**RBR**	0	1	0	0	1	0	1
**SCF**	0	0	0	0	1	0	1
**CYCD3;1**	0	1	1	1	1	0	1
Freq.	100%						

CYCA2;3 gain of function

Attractor(s) with period 11											
	Attr. 1										
**APC/C**	1	1	0	0	0	0	0	0	0	0	1
**KRP1**	0	1	0	0	0	0	1	0	0	0	0
**CYCA2;3**	1	1	1	1	1	1	1	1	1	1	1
**CDKB1;1**	1	1	0	0	0	0	1	1	1	1	1
**CYCB1;1**	0	0	0	0	0	0	1	1	1	1	1
**MYB3R1/4**	1	0	0	0	0	0	1	1	1	1	1
**MYB77**	0	0	0	0	0	1	1	1	0	0	0
**E2Fe**	0	0	0	1	1	1	1	1	1	0	0
**E2Fc**	1	1	0	0	1	1	0	1	1	1	1
**E2Fb**	0	0	0	0	1	1	1	1	0	0	0
**E2Fa**	0	0	0	1	1	1	1	0	0	0	0
**RBR**	1	1	0	0	0	0	0	1	1	1	1
**SCF**	0	0	0	0	0	1	1	1	1	1	1
**CYCD3;1**	0	1	1	1	1	1	0	0	0	0	0
Freq.	100%										

CDKB1;1 loss of function

	Attractor(s) with period 7						
				Attr. 1			
**APC/C**	0	1	0	0	0	0	0
**KRP1**	0	0	0	1	1	1	1
**CYCA2;3**	0	0	0	1	1	1	1
**CDKB1;1**	0	0	0	0	0	0	0
**CYCB1;1**	0	0	0	1	1	1	1
**MYB3R1/4**	0	0	0	1	0	1	0
**MYB77**	0	0	1	0	1	0	0
**E2Fe**	0	1	1	1	1	1	1
**E2Fc**	0	1	0	1	1	0	1
**E2Fb**	0	1	0	1	1	0	1
**E2Fa**	1	1	1	1	1	1	1
**RBR**	0	1	0	0	1	0	1
**SCF**	0	0	0	0	1	0	1
**CYCD3;1**	0	1	1	1	1	0	1
Freq.				100%			

CDKB1;1 gain of function

	Attractor(s) with period 11										
	Attr. 1										
**APC/C**	1	1	0	0	0	0	0	0	0	0	1
**KRP1**	0	1	0	0	0	0	1	0	0	0	0
**CYCA2;3**	0	0	0	0	0	0	1	1	1	1	1
**CDKB1;1**	1	1	1	1	1	1	1	1	1	1	1
**CYCB1;1**	0	0	0	0	0	0	1	1	1	1	1
**MYB3R1/4**	1	0	0	0	0	0	1	1	1	1	1
**MYB77**	0	0	0	0	0	1	1	1	0	0	0
**E2Fe**	0	0	0	1	1	1	1	1	1	0	0
**E2Fc**	1	1	0	0	1	1	0	1	1	1	1
**E2Fb**	0	0	0	0	1	1	1	1	0	0	0
**E2Fa**	0	0	0	1	1	1	1	0	0	0	0
**RBR**	1	1	0	0	0	0	0	1	1	1	1
**SCF**	0	0	0	0	0	1	1	1	1	1	1
**CYCD3;1**	0	1	1	1	1	1	0	0	0	0	0
Freq.	100%										

CYCB1;1 loss of function

	Attractor(s) with period 8							
	Attr. 1							
**APC/C**	0	0	0	0	0	0	0	0
**KRP1**	0	0	0	0	1	0	0	0
**CYCA2;3**	0	0	0	0	1	1	1	1
**CDKB1;1**	0	0	0	0	1	1	1	1
**CYCB1;1**	0	0	0	0	0	0	0	0
**MYB3R1/4**	0	0	0	0	1	1	1	0
**MYB77**	0	0	0	1	1	1	0	0
**E2Fe**	0	1	1	1	1	1	1	0
**E2Fc**	0	0	1	1	0	1	1	1
**E2Fb**	0	0	1	1	1	1	0	0
**E2Fa**	0	1	1	1	1	0	0	0
**RBR**	0	0	0	0	0	1	1	1
**SCF**	0	0	0	1	1	1	1	1
**CYCD3;1**	0	1	1	1	0	0	0	0
Freq.	100%							

CYCB1;1 gain of function

	Attractor(s) with period 12											
	Attr. 1											
**APC/C**	1	1	1	0	0	0	0	0	0	0	0	1
**KRP1**	0	1	1	0	0	0	0	1	0	0	0	0
**CYCA2;3**	0	0	0	0	0	0	0	1	1	1	1	1
**CDKB1;1**	1	1	1	0	0	0	0	1	1	1	1	1
**CYCB1;1**	1	1	1	1	1	1	1	1	1	1	1	1
**MYB3R1/4**	1	1	0	0	0	0	0	1	1	1	1	1
**MYB77**	0	0	0	0	0	0	1	1	1	0	0	0
**E2Fe**	0	0	0	0	1	1	1	1	1	1	0	0
**E2Fc**	1	1	1	0	0	1	1	0	1	1	1	1
**E2Fb**	0	0	0	0	0	1	1	1	1	0	0	0
**E2Fa**	0	0	0	0	1	1	1	1	0	0	0	0
**RBR**	1	1	1	0	0	0	0	0	1	1	1	1
**SCF**	0	0	0	0	0	0	1	1	1	1	1	1
**CYCD3;1**	0	1	1	1	1	1	1	0	0	0	0	0
Freq.	100%											

MYB3R1/4 loss of function

Attractor(s) with period 3			
	Attr. 1		
**APC/C**	0	0	0
**KRP1**	1	0	0
**CYCA2;3**	1	0	0
**CDKB1;1**	1	0	0
**CYCB1;1**	1	0	0
**MYB3R1/4**	0	0	0
**MYB77**	0	0	1
**E2Fe**	1	1	1
**E2Fc**	0	1	0
**E2Fb**	0	1	0
**E2Fa**	1	0	0
**RBR**	0	1	0
**SCF**	0	0	1
**CYCD3;1**	0	1	1
Freq.	17.87%		

Attractor(s) with period 7							
	Attr. 2						
**APC/C**	0	0	0	0	0	0	0
**KRP1**	0	0	0	0	0	1	0
**CYCA2;3**	1	0	0	0	0	1	1
**CDKB1;1**	1	0	0	0	0	1	1
**CYCB1;1**	1	0	0	0	0	1	1
**MYB3R1/4**	0	0	0	0	0	0	0
**MYB77**	0	0	0	0	1	1	1
**E2Fe**	1	1	1	1	1	1	1
**E2Fc**	0	0	0	1	1	0	1
**E2Fb**	0	0	0	1	1	1	1
**E2Fa**	0	0	1	1	1	1	0
**RBR**	0	0	0	0	0	0	1
**SCF**	0	0	0	0	1	1	1
**CYCD3;1**	0	1	1	1	1	0	0
Freq.	82.13%						

MYB3R1/4 gain of function

	Fixed-point attractor(s)	
	Attr. 1	Attr. 2
**APC/C**	1	1
**KRP1**	1	1
**CYCA2;3**	0	0
**CDKB1;1**	1	1
**CYCB1;1**	0	0
**MYB3R1/4**	1	1
**MYB77**	0	0
**E2Fe**	0	0
**E2Fc**	1	1
**E2Fb**	0	0
**E2Fa**	0	1
**RBR**	1	1
**SCF**	0	0
**CYCD3;1**	1	1
Freq.	98.61%	1.39%

MYB77 loss of function

	Attractor(s) with period 7						
				Attr. 1			
**APC/C**	0	1	0	0	0	0	0
**KRP1**	0	0	0	0	0	0	0
**CYCA2;3**	0	0	0	0	0	0	0
**CDKB1;1**	0	0	0	0	0	0	1
**CYCB1;1**	0	0	0	0	0	0	1
**MYB3R1/4**	0	0	0	0	0	0	0
**MYB77**	0	0	0	0	0	0	0
**E2Fe**	0	1	1	1	1	1	1
**E2Fc**	0	1	0	1	1	0	1
**E2Fb**	0	1	0	1	1	1	1
**E2Fa**	1	1	1	1	1	1	1
**RBR**	0	1	0	0	0	0	1
**SCF**	0	0	0	0	1	1	1
**CYCD3;1**	0	1	1	1	1	0	0
Freq.				100%			

MYB77 gain of function

	Fixed-point attractor(s)
	Attr. 1
**APC/C**	0
**KRP1**	0
**CYCA2;3**	1
**CDKB1;1**	1
**CYCB1;1**	1
**MYB3R1/4**	1
**MYB77**	1
**E2Fe**	1
**E2Fc**	1
**E2Fb**	0
**E2Fa**	0
**RBR**	1
**SCF**	1
**CYCD3;1**	0
Freq.	100%

E2Fe loss of function

Attractor(s) with period 7

	Attr. 1						
**APC/C**	0	1	1	1	1	1	1
**KRP1**	0	0	0	1	1	1	1
**CYCA2;3**	0	0	0	0	0	0	0
**CDKB1;1**	0	0	0	1	1	1	1
**CYCB1;1**	0	0	0	0	0	0	0
**MYB3R1/4**	0	0	0	1	1	1	0
**MYB77**	0	0	1	1	1	0	0
**E2Fe**	0	0	0	0	0	0	0
**E2Fc**	0	1	1	1	1	1	1
**E2Fb**	0	1	1	1	1	0	0
**E2Fa**	1	1	1	1	1	1	1
**RBR**	0	0	0	0	1	1	1
**SCF**	0	0	0	0	0	0	0
**CYCD3;1**	1	1	1	1	1	1	1
Freq.	100%						

E2Fe gain of function

	Fixed-point attractor(s)
	Attr. 1
**APC/C**	0
**KRP1**	0
**CYCA2;3**	1
**CDKB1;1**	1
**CYCB1;1**	1
**MYB3R1/4**	1
**MYB77**	0
**E2Fe**	1
**E2Fc**	1
**E2Fb**	0
**E2Fa**	0
**RBR**	1
**SCF**	1
**CYCD3;1**	0
Freq.	100%

E2Fc loss of function

	Fixed-point attractor(s)
	Attr. 1
**APC/C**	0
**KRP1**	0
**CYCA2;3**	1
**CDKB1;1**	1
**CYCB1;1**	1
**MYB3R1/4**	1
**MYB77**	0
**E2Fe**	1
**E2Fc**	0
**E2Fb**	0
**E2Fa**	0
**RBR**	1
**SCF**	1
**CYCD3;1**	0
Freq.	100%

E2Fc gain of function

	Fixed-point attractor(s)
	Attr. 1
**APC/C**	0
**KRP1**	0
**CYCA2;3**	0
**CDKB1;1**	0
**CYCB1;1**	0
**MYB3R1/4**	0
**MYB77**	0
**E2Fe**	0
**E2Fc**	1
**E2Fb**	0
**E2Fa**	0
**RBR**	0
**SCF**	0
**CYCD3;1**	1
Freq.	100%

E2Fb loss of function

	Fixed-point attractor(s)
	Attr. 1
**APC/C**	1
**KRP1**	0
**CYCA2;3**	0
**CDKB1;1**	0
**CYCB1;1**	0
**MYB3R1/4**	0
**MYB77**	0
**E2Fe**	0
**E2Fc**	1
**E2Fb**	0
**E2Fa**	1
**RBR**	0
**SCF**	0
**CYCD3;1**	1
Freq.	100%

E2Fb gain of function

Attractor(s) with period 9									
	Attr. 1								
**APC/C**	1	1	0	0	0	0	0	0	1
**KRP1**	0	1	0	0	1	0	0	0	0
**CYCA2;3**	0	0	0	0	1	1	1	1	1
**CDKB1;1**	1	1	0	1	1	1	1	1	1
**CYCB1;1**	0	0	0	1	1	1	1	1	1
**MYB3R1/4**	1	0	0	0	1	1	1	1	1
**MYB77**	0	0	0	1	1	1	0	0	0
**E2Fe**	0	0	0	1	1	1	1	0	0
**E2Fc**	1	1	0	0	0	1	1	1	1
**E2Fb**	1	1	1	1	1	1	1	1	1
**E2Fa**	0	0	0	1	1	0	0	0	0
**RBR**	1	1	0	0	0	1	1	1	1
**SCF**	0	0	0	1	1	1	1	1	1
**CYCD3;1**	0	1	1	1	0	0	0	0	0
Freq.	100%								

E2Fa loss of function

	Fixed-point attractor(s)
	Attr. 1
**APC/C**	0
**KRP1**	0
**CYCA2;3**	0
**CDKB1;1**	0
**CYCB1;1**	0
**MYB3R1/4**	0
**MYB77**	0
**E2Fe**	1
**E2Fc**	0
**E2Fb**	0
**E2Fa**	0
**RBR**	0
**SCF**	0
**CYCD3;1**	1
Freq.	100%

E2Fa gain of function

Attractor(s) with period 8

	Attr. 1							
**APC/C**	0	1	0	0	0	0	0	0
**KRP1**	0	0	0	1	1	0	1	0
**CYCA2;3**	0	0	0	1	1	1	1	1
**CDKB1;1**	0	0	0	1	1	1	1	1
**CYCB1;1**	0	0	0	1	1	1	1	1
**MYB3R1/4**	0	0	0	1	0	1	1	0
**MYB77**	0	0	1	0	1	0	0	0
**E2Fe**	0	1	1	1	1	1	1	0
**E2Fc**	0	1	0	1	1	0	1	1
**E2Fb**	0	1	0	1	1	0	1	0
**E2Fa**	1	1	1	1	1	1	1	1
**RBR**	0	1	0	0	1	0	1	1
**SCF**	0	0	0	0	1	0	1	1
**CYCD3;1**	0	1	1	1	1	0	1	0
Freq.	59.52%							

Attractor(s) with period 10

	Attr. 2									
**APC/C**	1	1	0	1	0	0	0	0	0	1
**KRP1**	0	1	0	0	0	1	0	0	0	0
**CYCA2;3**	0	0	0	0	0	1	1	1	1	1
**CDKB1;1**	1	1	0	0	0	1	1	1	1	1
**CYCB1;1**	0	0	0	0	0	1	1	1	1	1
**MYB3R1/4**	1	0	0	0	0	1	1	1	1	1
**MYB77**	0	0	0	0	1	1	1	0	0	0
**E2Fe**	0	0	0	1	1	1	1	1	0	0
**E2Fc**	1	1	0	1	1	1	1	1	1	1
**E2Fb**	0	0	0	1	1	1	1	0	0	0
**E2Fa**	1	1	1	1	1	1	1	1	1	1
**RBR**	1	1	0	0	0	0	1	1	1	1
**SCF**	0	0	0	0	0	1	1	1	1	1
**CYCD3;1**	0	1	1	1	1	1	0	0	0	0
Freq.	40.48%									

RBR loss of function

Attractor(s) with period 3			
	Attr. 1		
**APC/C**	0	0	0
**KRP1**	1	1	0
**CYCA2;3**	1	1	0
**CDKB1;1**	1	1	0
**CYCB1;1**	1	1	0
**MYB3R1/4**	1	0	0
**MYB77**	0	0	1
**E2Fe**	1	1	1
**E2Fc**	0	1	0
**E2Fb**	0	1	0
**E2Fa**	1	0	0
**RBR**	0	0	0
**SCF**	0	1	1
**CYCD3;1**	0	1	0
Freq.	18.02%		

Attractor(s) with period 12												
	Attr. 2											
**APC/C**	1	1	0	0	0	0	0	0	0	0	0	1
**KRP1**	0	1	0	0	0	0	1	0	0	0	0	0
**CYCA2;3**	0	0	0	0	0	0	1	1	1	1	1	1
**CDKB1;1**	1	1	0	0	0	0	1	1	1	1	1	1
**CYCB1;1**	0	0	0	0	0	0	1	1	1	1	1	1
**MYB3R1/4**	1	0	0	0	0	0	1	1	1	1	1	1
**MYB77**	0	0	0	0	0	1	1	1	1	0	0	0
**E2Fe**	0	0	0	1	1	1	1	1	1	1	0	0
**E2Fc**	1	1	0	0	1	1	0	1	1	1	1	1
**E2Fb**	0	0	0	0	1	1	1	1	0	0	0	0
**E2Fa**	0	0	0	1	1	1	1	0	0	0	0	0
**RBR**	0	0	0	0	0	0	0	0	0	0	0	0
**SCF**	0	0	0	0	0	1	1	1	1	1	1	1
**CYCD3;1**	0	1	1	1	1	1	0	0	0	0	0	0
Freq.	81.98%											

RBR gain of function

	Fixed-point attractor(s)
	Attr. 1
**APC/C**	0
**KRP1**	0
**CYCA2;3**	0
**CDKB1;1**	0
**CYCB1;1**	0
**MYB3R1/4**	0
**MYB77**	0
**E2Fe**	1
**E2Fc**	0
**E2Fb**	0
**E2Fa**	1
**RBR**	1
**SCF**	0
**CYCD3;1**	1
Freq.	100%

SCF loss of function

Attractor(s) with period 8								
	Attr. 1							
**APC/C**	0	0	0	0	0	0	0	0
**KRP1**	0	0	0	0	1	1	1	1
**CYCA2;3**	0	0	0	0	1	1	1	1
**CDKB1;1**	0	0	0	0	1	1	1	1
**CYCB1;1**	0	0	0	0	1	1	1	1
**MYB3R1/4**	0	0	0	0	1	1	1	0
**MYB77**	0	0	0	1	1	1	0	0
**E2Fe**	0	1	1	1	1	1	1	0
**E2Fc**	0	0	1	1	1	1	1	1
**E2Fb**	0	0	1	1	1	1	0	0
**E2Fa**	0	1	1	1	1	0	0	0
**RBR**	0	0	0	0	0	1	1	1
**SCF**	0	0	0	0	0	0	0	0
**CYCD3;1**	1	1	1	1	1	1	1	1
Freq.	100%							

SCF gain of function

	Attractor(s) with period 2	
	Attr. 1	
**APC/C**	0	1
**KRP1**	0	0
**CYCA2;3**	0	0
**CDKB1;1**	0	0
**CYCB1;1**	0	0
**MYB3R1/4**	0	0
**MYB77**	0	0
**E2Fe**	0	1
**E2Fc**	0	1
**E2Fb**	0	1
**E2Fa**	1	1
**RBR**	0	1
**SCF**	1	1
**CYCD3;1**	0	0
Freq.	100%	

CYCD3;1 loss of function

	Attractor(s) with period 2	
	Attr. 1	
**APC/C**	0	1
**KRP1**	0	0
**CYCA2;3**	0	0
**CDKB1;1**	0	0
**CYCB1;1**	0	0
**MYB3R1/4**	0	0
**MYB77**	0	0
**E2Fe**	0	1
**E2Fc**	0	1
**E2Fb**	0	1
**E2Fa**	1	1
**RBR**	0	1
**SCF**	0	0
**CYCD3;1**	0	0
Freq.	100%	

CYCD3;1 gain of function

	Fixed-point attractor(s)
	Attr. 1
**APC/C**	0
**KRP1**	0
**CYCA2;3**	1
**CDKB1;1**	1
**CYCB1;1**	1
**MYB3R1/4**	1
**MYB77**	0
**E2Fe**	1
**E2Fc**	0
**E2Fb**	0
**E2Fa**	0
**RBR**	0
**SCF**	1
**CYCD3;1**	1
Freq.	100%
